# Screening for Chemical Characterization and Pharmacological Properties of Different Extracts from *Nepeta italica*

**DOI:** 10.3390/plants12152785

**Published:** 2023-07-27

**Authors:** Alessandra Acquaviva, Simonetta Cristina Di Simone, Nilofar, Abdelhakim Bouyahya, Gokhan Zengin, Lucia Recinella, Sheila Leone, Luigi Brunetti, Abdullahi Ibrahim Uba, Osman Guler, Maruf Balos, Ugur Cakilcioğlu, Luigi Menghini, Claudio Ferrante, Giustino Orlando, Maria Loreta Libero, Annalisa Chiavaroli

**Affiliations:** 1 Department of Pharmacy, G. d’Annunzio University of Chieti-Pescara, 66100 Chieti, Italy; alessandra.acquaviva@unich.it (A.A.); simonetta.disimone@unich.it (S.C.D.S.); nilofar.nilofar@unich.it (N.); lucia.recinella@unich.it (L.R.); sheila.leone@unich.it (S.L.); luigi.brunetti@unich.it (L.B.); luigi.menghini@unich.it (L.M.); claudio.ferrante@unich.it (C.F.); giustino.orlando@unich.it (G.O.); annalisa.chiavaroli@unich.it (A.C.); 2 Laboratory of Human Pathologies Biology, Faculty of Sciences, Department of Biology, Mohammed V University in Rabat, Rabat 10106, Morocco; a.bouyahya@um5r.ac.ma; 3 Physiology and Biochemistry Laboratory, Department of Biology, Science Faculty, Selcuk University, Konya 42130, Turkey; 4 Department of Molecular Biology and Genetics, Istanbul AREL University, Istanbul 34537, Turkey; abdullahi.iu2@gmail.com; 5 Pertek Sakine Genç Vocational School, Munzur University, Pertek 62500, Turkey; osmanguler@munzur.edu.tr (O.G.); ucakilcioglu@yahoo.com (U.C.); 6 Sanlıurfa Provincial Directorate of National Education, Sanlıurfa 63320, Turkey; mbalos@gmail.com

**Keywords:** *Nepeta italica*, antioxidant, rosmarinic acid, anti-inflammatory

## Abstract

Plants from the *Nepeta* genus have been proved to possess different pharmacological properties, among which are antimicrobial, antioxidant, anti-inflammatory, analgesic, and cytotoxic effects. *Nepeta italica* is a medicinal plant traditionally used for its analgesic effects, and in the present study, the phytochemical composition and biological effects of hexane, dichloromethane (DCM), ethyl acetate (EA), ethanol, ethanol-water, and water extracts of the aerial parts were investigated for determining phenolic composition, antioxidant effects, and anti-inflammatory effects in isolated mouse colon specimens exposed to lipopolysaccharide (LPS). Polar extracts were the richest in terms of phenolic compounds, especially rosmarinic acid. In parallel, ethanol, ethanol-water, and water extracts were also the most effective as scavenging/reducing and enzyme inhibition agents, especially towards cholinesterases and α-glucosidase, and in inhibiting the LPS-induced cyclooxygenase-2 (COX-2) and tumor necrosis factor α (TNFα) gene expression in mouse colon. This poses the basis for future in vivo investigations for confirming the protective effects of polar extracts of *N. italica* against inflammatory bowel diseases.

## 1. Introduction

Medicinal plants have long been considered as a precious source of simple medicines (*medicamenta simplex*) above all for their content of specialized metabolites that may exert health-promoting effects through binding interactions with specific human protein targets [1].

Plants belonging to the genus *Nepeta* (about 280 species distributed across Europe, Asia, North America, and Africa) have been long used for treating different disorders due to numerous pharmacological properties, among which are antimicrobial, antioxidant, anti-inflammatory, analgesic, and cytotoxicity against cancer cells [2].

Such effects could be related, albeit in part, to the content of specialized metabolites in the plant phytocomplex, which includes monoterpenes, sesquiterpenes, unsaturated lactones, and phenolic compounds [1], although studies have mostly considered the activities and composition of the essential oil. Decoctions and infusions of *Nepeta* species have been traditionally used as sedatives, antioxidants, and anti-inflammatories [3].

*Nepeta italica* L. is 1 of the 33 species represented in Turkey, and the essential oil was previously investigated for its analgesic effect [4], whereas different polarity extracts from the aerial parts of the subsp. *cadmea* were effective as antioxidant and anthelmintic agents [5]. Considering the potential anti-inflammatory effects related to the use of the aerial parts of *Nepeta* species [6], in the present study, the phytochemical composition and biological effects of hexane, dichloromethane (DCM), ethyl acetate (EA), ethanol, ethanol-water, and water extracts of the aerial parts of *N. italica* were investigated for unravelling potential applications against inflammatory bowel diseases (IBDs), whose etiology is related to increased oxidative stress [7]. IBDs represent a sensitive challenge in pharmacotherapy [8], and herbal extracts with antioxidant/anti-inflammatory effects may be effective for contrasting IBD symptoms. For this purpose, the extracts of *N. italica* were tested for scavenging/reducing properties and enzyme inhibition effects in free cell models. Phenolic composition was in parallel determined via both colorimetric and chromatographic analyses. Finally, the protective effects of the extracts, measured as inhibition of COX-2 and TNFα gene expression, were assayed in an ex vivo experimental model of colon inflammation, constituted by isolated mouse colon specimens challenged with lipopolysaccharide (LPS) [9].

## 2. Results

### 2.1. Total Phenolic and Flavonoid Content

The results obtained regarding the total phenolic and flavonoid contents of different extracts of *Nepeta italica* are presented in Table 1. The n-hexane extract revealed a total phenolic content of 18.46 ± 0.23 mg GAE/g and a total flavonoid content of 15.66 ± 0.46 mg RE/g. The dichloromethane extract showed a similar total phenolic content (17.04 ± 0.16 mg GAE/g), but a slightly higher total flavonoid content (20.11 ± 0.37 mg RE/g). The ethyl acetate extract exhibited the highest total phenolic content among all tested extracts, with a value of 21.10 ± 1.10 mg GAE/g, while the total flavonoid content was 17.45 ± 0.20 mg RE/g. The ethanol and ethanol-water extracts also showed significant total phenolic contents, with values of 28.41 ± 0.11 mg GAE/g and 44.07 ± 0.91 mg GAE/g, respectively. However, the total flavonoid content of the ethanol extract (15.28 ± 0.48 mg RE/g) was higher than the one of the ethanol-water extract (8.47 ± 0.12 mg RE/g). Finally, the aqueous extract revealed the highest total phenolic content (59.13 ± 0.23 mg GAE/g), but a relatively low total flavonoid content (11.46 ± 0.45 mg RE/g).

The results obtained for the total phenolic and flavonoid contents of *N. italica* extracts exhibit significant variations depending on the solvent used for extraction.

Extracts obtained with organic solvents such as n-hexane and dichloromethane showed relatively lower total phenolic contents compared to the other extracts. This could be attributed to the properties of these solvents, which have a low affinity for phenolic compounds [10,11]. However, the dichloromethane extract exhibited a slightly higher total flavonoid content compared to the other tested organic solvents [12,13]. This suggests that specific flavonoids present in *N. italica* may be more soluble in dichloromethane.

On the other hand, the ethyl acetate extract exhibited the highest total phenolic content among all the tested extracts. Ethyl acetate is an intermediate polarity solvent, which enables efficient extraction of phenolic compounds present in the plant [14,15]. Additionally, it also showed a relatively high total flavonoid content, indicating its ability to effectively extract a wide range of bioactive compounds from *N. italica* [16,17].

The extracts obtained from ethanol and ethanol-water also revealed significant total phenolic contents. Ethanol is commonly used as a solvent for extracting phenolic compounds due to its solubility and ability to extract various bioactive compounds [18,19,20]. The ethanol-water extract showed a higher total phenolic content than the ethanol extract, suggesting that the use of a solvent mixture can enhance the extraction efficiency of phenolics from *N. italica* [16,21].

Finally, the aqueous extract exhibited the highest total phenolic content among all the tested extracts. Water is a polar and universally available solvent, making it an attractive option for the extraction of bioactive compounds [22,23,24]. However, the total flavonoid content of the aqueous extract was relatively low compared to the other solvents.

In conclusion, the results clearly indicate that the choice of extraction solvent plays a crucial role in the quantity and type of phenolic and flavonoid compounds extracted from *N. italica*. Ethyl acetate has proven to be the most effective solvent for extracting total phenols, while the aqueous extract showed the highest total phenolic content.

### 2.2. Chemical Composition

**Figure 1:**
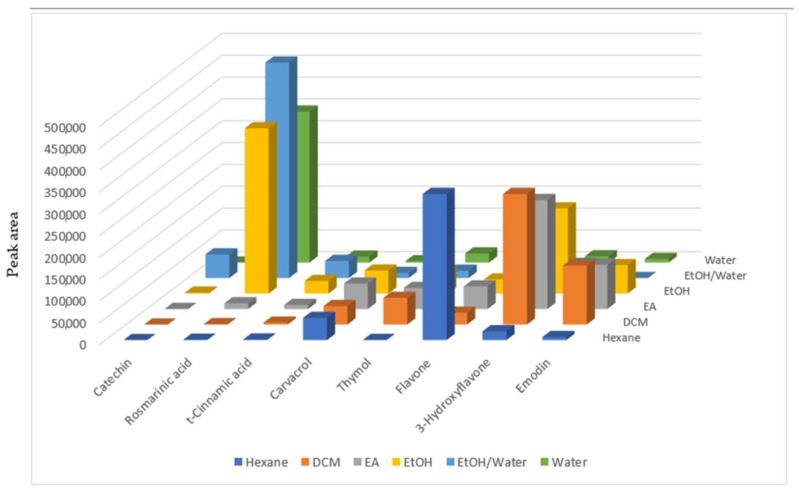
Phenolic profile of the *Nepeta italica* extracts.

The results of the chemical composition of *N. italica* extracts are presented in Figure 1 and Figure S1 and Table S2. The analysis revealed the presence of several compounds in *N. italica* extracts. Gallic acid was detectable only in the aqueous and ethanol-water extracts. 3-hydroxytyrosol, a phenolic compound, was present in the dichloromethane, ethyl acetate, ethanol, and ethanol-water extracts. Caffeic acid, another phenolic compound, was present in the dichloromethane, ethyl acetate, ethanol, and ethanol-water extracts. Catechin, a flavonoid, was detectable only in the dichloromethane extract and the ethanol extract. Other phenolic compounds such as genistic acid, loganic acid, chlorogenic acid, caffeic acid, and vanillic acid were present in the ethanol extract but not detectable in other extracts.

Epicatechin, a flavanol, was detectable in the n-hexane, dichloromethane, ethyl acetate, and ethanol-water extracts, whilst other compounds such as syringic acid, syringaldehyde, p-coumaric acid, t-ferulic acid, rutin, hyperoside, resveratrol, rosmarinic acid, trans-cinnamic acid, quercetin, naringenin, 2,3-dimethylbenzoic acid, hesperetin, and kaempferol were all present in the polar extracts. By contrast, the phenolic monoterpenes carvacrol and thymol were present at higher levels in the lipophilic extracts.

The results of the chemical composition of *N. italica* extracts revealed a diversity of phenolic and flavonoid compounds present in the plant. These compounds are known for their antioxidant properties and beneficial effects on health.

Gallic acid, detected in the aqueous and ethanol-water extracts, is a powerful antioxidant that can contribute to protection against free radical damage in the body [16]. Its presence in these extracts suggests that the use of polar solvents such as water and ethanol can be effective for extracting this acid. 3-hydroxytyrosol, present in several extracts, is also a potentially beneficial antioxidant for cardiovascular health [25]. Its varying concentrations in different extracts indicate that the choice of extraction solvent can influence its content. For example, the ethanol extract showed a relatively high content of 3-hydroxytyrosol, suggesting that ethanol can be an effective solvent for extracting this compound.

Caffeic acid, another phenolic compound detected in multiple extracts, is known for its anti-inflammatory and antioxidant properties [26,27]. Its presence in the dichloromethane, ethyl acetate, ethanol, and ethanol-water extracts suggests that these solvents may be suitable for extracting this acid. Catechin, a flavonoid, was present in the dichloromethane extract and the ethanol extract at high concentrations [28,29]. Catechin is widely studied for its antioxidant properties and its potential effects on the prevention of various diseases.

Other phenolic and flavonoid compounds, such as genistic acid, chlorogenic acid, caffeic acid, rosmarinic acid, and quercetin, were detected in certain extracts, showcasing the richness and diversity of compounds present in *N. italica*.

Indeed, it is important to note that some compounds were not detected in certain extracts, suggesting that their extraction may be more effective with specific solvents. These results emphasize the significant influence of the choice of extraction solvent on the chemical composition of *N. italica* extracts. The results of this study highlight the presence of diverse phenolic and flavonoid compounds in *N. italica* extracts, demonstrating its potential as a rich source of bioactive compounds. These findings can contribute to a better understanding of the pharmacological properties and potential applications of *N. italica* in the field of health and nutrition.

### 2.3. Antioxidant Properties

Table 2 presents the antioxidant properties of *N. italica* extracts, quantified as milligrams of trolox equivalent per gram of extract (mg TE/g) for DPPH, ABTS, CUPRAC, and FRAP, millimoles of BHT equivalent per gram of extract (mmol TE/g) for PBD, and milligrams of EDTA equivalent per gram of extract (mg EDTAE/g) for MCA. The results consistently indicate significant antioxidant activity in all tested methods for all *N. italica* extracts. In ABTS and DPPH assays, ethanol/water and water extracts were found to have the best scavenging ability, followed by ethanol and ethyl acetate extracts. With respect to the reducing power tests, the water extract showed the strongest ability (CUPRAC: 208.59 mg TE/g; FRAP: 116.86 mg TE/g), while the weakest ability was found in the n-hexane extract (CUPRAC: 37.03 mg TE/g; FRAP: 19.33 mg TE/g). However, the total antioxidant activity in the phosphmolybdenum assay can be ranked as follows: ethyl acetate > n-hexane > dichloromethane > ethanol > ethanol/water > water. For metal chelating ability, the n-hexane extract had the best effect, and the dichloromethane, ethyl acetate, and water extracts showed similar ability (*p* > 0.05).

The findings demonstrated that the n-hexane extract of *N. italica* possesses moderate antioxidant activity. While its values are slightly lower compared to other extracts, it still showcases notable antioxidant capabilities. This suggests that the n-hexane extract could be a suitable option for applications where a moderate level of antioxidant activity is desired. On the other hand, the dichloromethane extract exhibits higher antioxidant activity compared to the n-hexane extract. The significantly higher values obtained for DPPH, ABTS, CUPRAC, FRAP, PBD, and MCA indicated the presence of potent antioxidant compounds in this extract. Thereby, the dichloromethane extract of *N. italica* could be a promising candidate for industries requiring strong antioxidant properties [19,30]. Moreover, the ethyl acetate extract demonstrates even higher antioxidant activity, surpassing both the n-hexane and dichloromethane extracts. The substantial values obtained for all tested methods indicate a robust antioxidant capacity. This high antioxidant activity could be attributed to the presence of phenolic compounds and flavonoids in the ethyl acetate extract. Therefore, this extract holds great potential for applications where a strong antioxidant effect is desired [31,32]

Remarkably, the ethanol extract of *N. italica* displays the highest antioxidant activity among all the tested extracts. The significantly elevated values obtained for DPPH, ABTS, CUPRAC, FRAP, PBD, and MCA highlight the exceptional antioxidant capacity of this extract. Previous studies indicated that the ethanol extract could be particularly valuable in industries requiring potent antioxidant properties, such as the development of antioxidant-rich supplements or functional food products [33,34].

In conclusion, the study revealed that *N. italica* extracts possess significant antioxidant activities, with each extract displaying varying levels of potency. The diverse range of antioxidant properties observed in these extracts opens up opportunities for their utilization in various industries, including pharmaceuticals, food, and nutraceuticals. Further research can explore the identification and isolation of specific antioxidant compounds from these extracts to better understand their mechanisms of action and potential therapeutic applications.

### 2.4. Enzyme Inhibitory Effects

#### 2.4.1. In Vitro Antidiabetic Effects

The study of the inhibitory activity of *N. italica* extracts on amylase and glucosidase, two essential enzymes involved in carbohydrate digestion [35,36], has revealed fascinating results. These findings highlight significant variations in the inhibitory activity among the different tested extracts (Table 3).

Among the extracts, the one obtained with n-hexane solvent demonstrated a moderate inhibition activity against amylase, with a value of 0.37 mmol ACAE/g. However, its inhibitory activity against glucosidase was much more pronounced, reaching a remarkable value of 4.91 mmol ACAE/g. These results suggested that the n-hexane extract of *N. italica* exhibits a marked preference for inhibiting glucosidase, highlighting its promising potential for controlling carbohydrate digestion. Similarly, the extract obtained with dichloromethane solvent also manifested moderate inhibition activity against amylase, with a value of 0.58 mmol ACAE/g. In contrast, its inhibitory activity against glucosidase was lower, reaching only 0.14 mmol ACAE/g. These results revealed a stronger preference of the dichloromethane extract of *N. italica* for inhibiting amylase compared to glucosidase, thus emphasizing its specific potential for modulating carbohydrate digestion. On the other hand, the ethyl acetate extract demonstrated similar inhibition values for both amylase (0.51 mmol ACAE/g) and glucosidase (0.53 mmol ACAE/g). These results indicate a relatively balanced inhibitory activity of the ethyl acetate extract of *N. italica* towards both enzymes, revealing its versatile potential for regulating carbohydrate digestion. In contrast, the extract obtained with ethanol revealed moderate inhibition activity against amylase (0.33 mmol ACAE/g), but a significantly more pronounced inhibitory activity against glucosidase (5.38 mmol ACAE/g). These results suggested that the ethanol extract of *N. italica* exhibits an increased propensity to inhibit glucosidase compared to amylase, highlighting its potential interest for modulating carbohydrate digestion. The ethanol-water extract, on the other hand, demonstrated a marked preference for inhibiting glucosidase, with a high value of 5.60 mmol ACAE/g, while its inhibitory activity against amylase was moderate (0.25 mmol ACAE/g). These results highlighted the high potential of the ethanol-water extract of *N. italica* for specifically inhibiting glucosidase, suggesting a possible application in regulating carbohydrate-related metabolic responses. Finally, the aqueous extract of *N. italica* revealed low inhibitory activity against both enzymes, with values of 0.05 mmol ACAE/g for amylase and 0.94 mmol ACAE/g for glucosidase.

Multiple studies have been conducted to evaluate the inhibitory activity of *Nepeta* extracts on these enzymes. These studies have utilized various analysis and assay methods to measure the inhibitory activity. The results of these studies have shown that *Nepeta* genus extracts can exhibit significant inhibitory activity against amylase and glucosidase [37,38,39]. This implies that these extracts have the potential to slow down carbohydrate digestion and reduce glucose absorption, which can be beneficial for individuals with diabetes or those seeking to control their blood sugar levels.

These results underscored the importance of evaluating plant extracts for their specific inhibitory activity on digestive enzymes, offering promising perspectives in the development of new therapeutic approaches or dietary supplements for managing carbohydrate-related metabolic disorders.

#### 2.4.2. In Vitro Neuroprotective Effects

The extracts of *N. italica* were evaluated for their acetylcholinesterase (AChE) and butyrylcholinesterase (BChE) inhibition activity (Table 3), which are enzymes involved in the degradation of acetylcholine [40,41]. The present results provided valuable information on the potential of these extracts as therapeutic agents for the treatment of neurodegenerative diseases associated with cholinergic dysfunction. The n-hexane extract showed moderate AChE inhibition activity, with a value of 3.02 mg GALAE/g. This extract also exhibited a BChE inhibition activity of 1.88 mg GALAE/g. Although the values obtained for the n-hexane extract were slightly lower than those of the other extracts, they still indicate a significant capacity to inhibit cholinesterase enzymes. In contrast, the dichloromethane extract revealed higher activity, with values of 2.93 mg GALAE/g for AChE and 2.40 mg GALAE/g for BChE. These results suggest that this extract has a stronger ability to inhibit cholinesterase enzymes, which could be attributed to the presence of specific bioactive compounds. The ethyl acetate extract also demonstrated significant inhibition activity against AChE and BChE, with values of 2.69 mg GALAE/g and 1.79 mg GALAE/g, respectively. These results indicated promising inhibitory properties of this *N. italica* extract against cholinesterase enzymes. The ethanol extract revealed the highest inhibitory activity among all tested extracts. It exhibited high values of 2.88 mg GALAE/g for AChE and 4.01 mg GALAE/g for BChE. The findings clearly demonstrated that the ethanol extract of *N. italica* has a high potential for inhibiting cholinesterase enzymes, making it a promising candidate for the development of drugs against neurodegenerative diseases. The ethanol-water extract also showed significant inhibitory activity, with values of 2.80 mg GALAE/g for AChE and 1.24 mg GALAE/g for BChE. These results indicate that this extract may also play an important role in modulating cholinesterase activity. In contrast, the aqueous extract exhibited very weak AChE inhibition activity (0.04 mg GALAE/g), and no BChE inhibition activity was observed. This suggests that the compounds present in this extract are not as effective at inhibiting cholinesterase enzymes as those in the other extracts.

Although studies on extracts of *N. italica* and their inhibitory activity against AChE and BChE are limited, some research on other species within the *Nepeta* genus suggested inhibitory potential. In this regard, Yilmaz et al. [42] isolated and characterized several terpenoids from *N. obtusicrena*, and they tested their inhibitory activity against AChE and BChE. The results showed that some of the studied terpenoids exhibited significant inhibitory activity against these enzymes. Additionally, Yilmaz et al. [43] isolated and identified a new compound from *N. sorgerae*, and they evaluated its potential inhibitory activity against AChE. In vitro tests were conducted to assess the inhibitory effects of the compound on the enzyme.

The findings of the study demonstrated that the isolated isopimarane diterpenoid exhibited significant inhibitory activity against AChE. This suggests that the compound has the potential to modulate acetylcholine levels in the brain by inhibiting AChE.

However, it should be noted that further research is needed to better understand the mechanisms of action and the potential effectiveness of *N. italica* extracts in inhibiting AChE and BChE. It is also important to emphasize that the inhibitory activity of these extracts may vary depending on various factors such as cultivation conditions, extraction methods, and plant parts used.

#### 2.4.3. In Vitro Dermatoprotective Effects

The activity of the extracts was evaluated in terms of tyrosinase inhibition, a key enzyme involved in melanin synthesis [44,45]. The results obtained revealed variable levels of inhibitory activity among the tested extracts (Table 3). Among the extracts, the n-hexane extract demonstrated the strongest tyrosinase inhibition activity, with a value of 72.12 mg KAE/g. This observation indicates a potent enzyme inhibitory potential, suggesting possible use in the field of skin pigmentation regulation. Furthermore, the dichloromethane extract also showed significant tyrosinase inhibition activity, with a value of 49.91 mg KAE/g. Although slightly lower than that of the n-hexane extract, this activity remains promising for potential application in cosmetic and dermatological products. On the other hand, the ethyl acetate extract displayed moderate tyrosinase inhibition activity, with a value of 56.29 mg KAE/g. This observation suggests its potential use in skin pigmentation regulation, although further studies are necessary to evaluate its effectiveness and identify the active compounds responsible for this activity. Similarly, the ethanol extract exhibited a tyrosinase inhibitory activity of 64.61 mg KAE/g, confirming its potential for regulating melanin synthesis. This significant activity highlights the interest in the ethanol extract of *N. italica* as a potential depigmenting agent for cosmetic applications. Regarding the ethanol-water extract, it also demonstrated significant tyrosinase inhibition activity, with a value of 59.52 mg KAE/g. This observation indicates promising inhibitory activity of this extract, deserving further investigation to assess its potential use in products aimed at regulating skin pigmentation. In contrast, the water extract showed the lowest tyrosinase inhibition activity among all tested extracts, with a value of 15.04 mg KAE/g. This low activity can be attributed to a reduced content of active compounds in the water extract of *N. italica*.

According to scientific studies, extracts of the *Nepeta* genus have shown inhibitory activity against tyrosinase. Kaska et al. [16] conducted in vitro tests to evaluate the ability of the extracts and essential oil to inhibit the activity of this enzyme. The results of the study revealed that extracts of *N. baytopii*, as well as its essential oil, exhibited significant tyrosinase inhibitory activity. This indicates that compounds present in the plant have the ability to reduce melanin production by inhibiting the activity of the tyrosinase enzyme. In another study conducted by Sharma et al. [19], they have provided evidence for the inhibitory activity of tyrosinase in specific species of the *Nepeta* genus. The presence of phytochemical compounds, including flavonoids and terpenoids, in these species has been shown to effectively regulate the activity of the tyrosinase enzyme.

These findings are promising, as they suggest that *Nepeta* species could be exploited in the development of cosmetic products aimed at regulating skin pigmentation. The use of these extracts or their active compounds could contribute to the development of treatments for skin pigmentation issues such as age spots or sunspots.

### 2.5. Ex Vivo Anti-Inflammatory Effects in the Colon

Inflammatory bowel diseases (IBDs) are chronic and relapsing colon pathologies related to an unbalanced intestinal immune response to external stimuli [7,46,47,48]. In this context, the increased pro-inflammatory biomarkers, among which are reactive oxygen/nitrogen (ROS/RNS) species, prostaglandins, and cytokines, reinforce the inflammatory status, thus leading to tissue damage [7]. Aminosalycilates, glucocorticoids, immune-suppressants, and tumor necrosis factor (TNF)α inhibitors are first-line pharmacological aids for IBDs. However, lack of efficacy and/or side effects are experienced by a wide plethora of patients (20–40%), thus making urgent the need for innovative therapies that may join efficacy to tolerability and reduction of side effects [9]. Medicinal plants have long been reported to possess the ability to challenge inflammation and oxidative stress pathways underlying IBDs [49,50]. Noteworthy is the potential to face colon inflammatory conditions through the use of home-made extracts, especially those prepared with traditional and biocompatible solvents (water, hydroalcoholic solutions) in the forms of infusions or decoctions. Such preparations could not only be effective and safe, but also represent smart and innovative strategies for implementing and valorizing local botanical resources and chains.

In the present study, the anti-inflammatory effects of *N. italica* extracts were evaluated in isolated colon specimens exposed to LPS in order to reproduce the burden of inflammation and oxidative stress occurring in IBDs in vivo.

**Figure 2:**
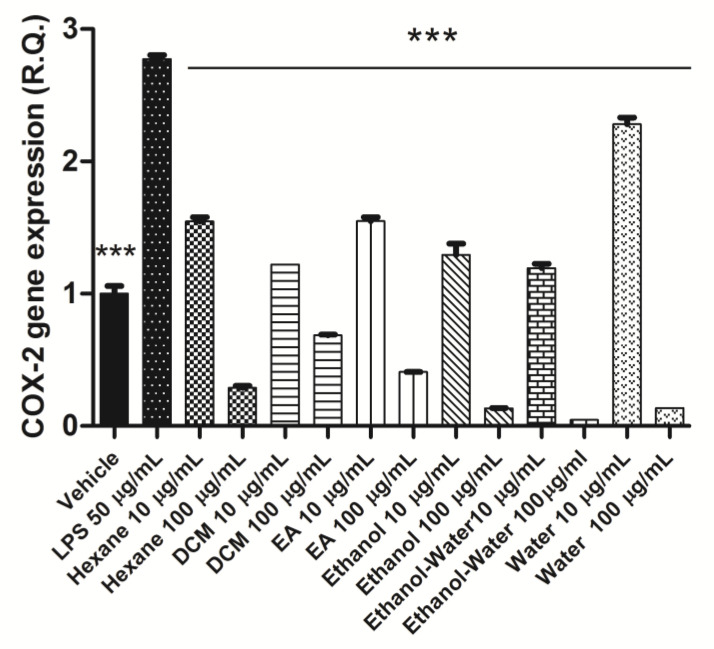
Inhibitory effect of *Nepeta italica* extracts on COX-2 gene expression in isolated mouse colon specimens exposed to LPS. ANOVA, *p* < 0.0001, *** *p* < 0.001 vs. LPS group.

**Figure 3:**
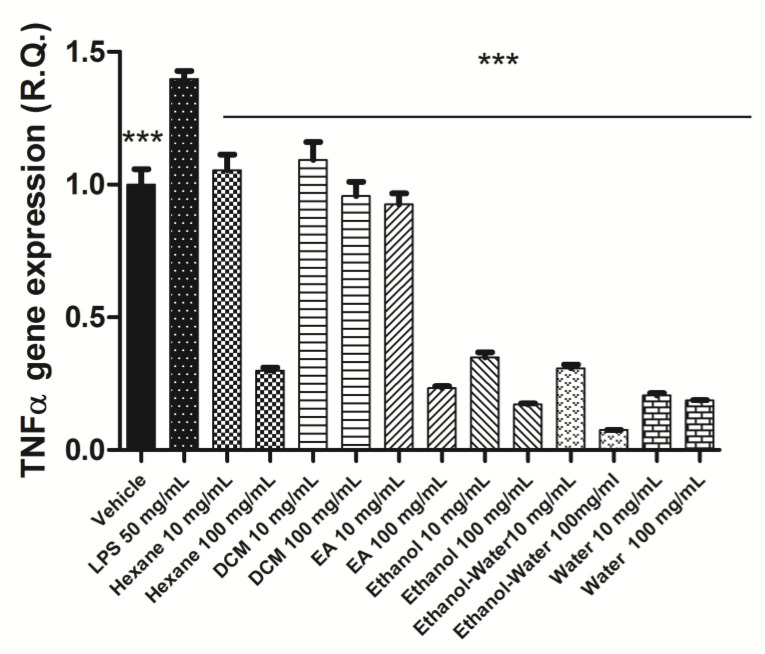
Inhibitory effect of *Nepeta italica* extracts on TNFα gene expression in isolated mouse colon specimens exposed to LPS. ANOVA, *p* < 0.0001, *** *p* < 0.001 vs. LPS group.

All extracts were effective in contrasting the LPS-induced up-regulation of both COX-2 and TNFα gene expression (Figure 2 and Figure 3). This could be related, albeit partially, to the content of phenolic compounds and the scavenging/reducing properties displayed by the extracts [51,52]. It is worthy to highlight the higher inhibitory effects on TNFα gene expression induced by ethanol, ethanol-water, and water extracts compared with the lipophilic extracts. We cannot exclude that the present results could be partially related to their higher content of rosmarinic acid, which displayed anti-inflammatory effects in experimental models of colon inflammation, including the capability to reduce the production of TNFα and COX-2. This poses the basis for future in vivo investigations for confirming the protective effects of polar extracts of *N. italica* against IBDs.

### 2.6. Molecular Docking

**Figure 4:**
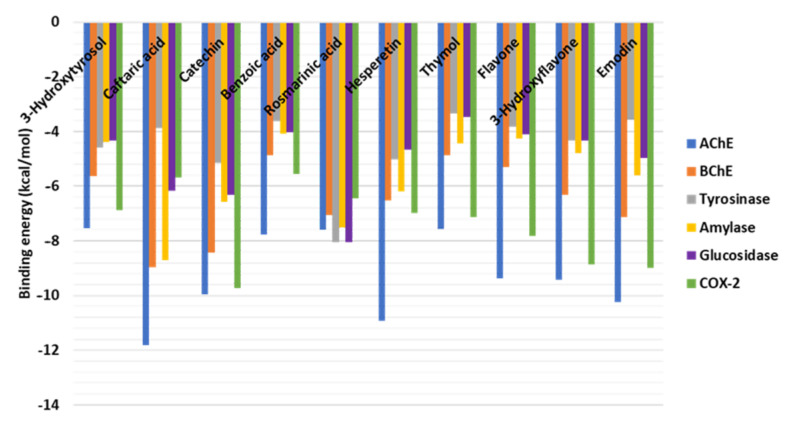
Docking score of the dominant compounds from different extracts from *Nepeta italica*.

**Figure 5:**
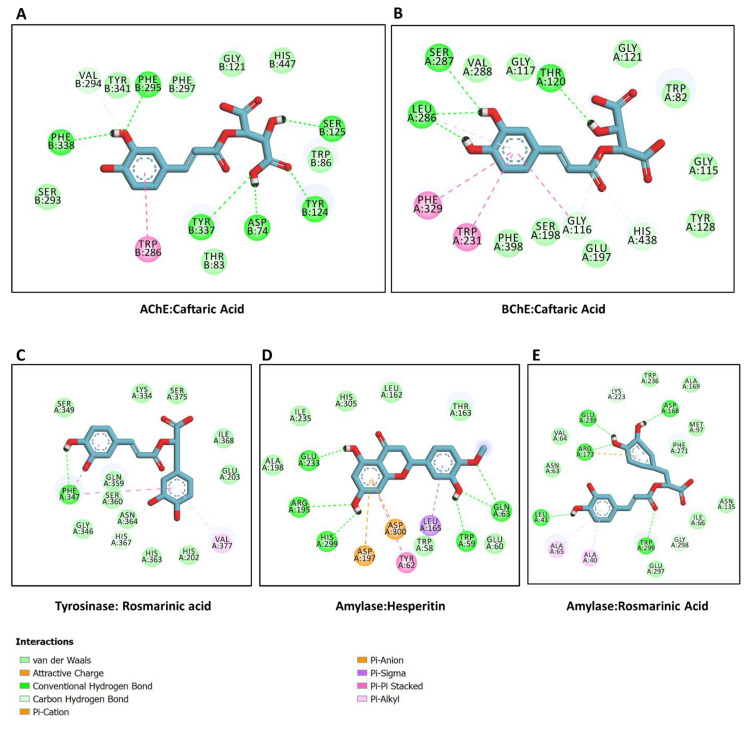
Interaction between target enzymes and the dominant bioactive compounds in different extracts from *Nepeta italica*: (**A**) AChE and caftaric acid, (**B**) BChE and caftaric acid, (**C**) tyrosinase and rosmarinic acid, (**D**) amylase and hesperitin, (**E**) glucosidase and rosmarinic acid.

This study provides an analysis of the binding mode and prediction of the binding propensity of each ligand against different target enzymes (Figure 4). The ligands bind to all the selected enzymes with different binding potentials. Caftaric acid bound to AChE (Figure 5A) and BChE (Figure 5B) in a similar mode, forming multiple H-bonds via hydroxyl groups and π–π stacked with amino acid residues lining the active sites. Notably, rosmarinic acid occupied the catalytic channel of tyrosinase with an interesting binding mode. Rosmarinic acid was buried in the narrow active site of tyrosinase mainly via an H-bond, two π–π stacked, and several van der Waals interactions (Figure 5C). The major interactions between amylase and hesperitin were multiple H-bonds formed near the entrance to, and deep inside, the active site, along with a couple of π–anion interactions (Figure 5D). Interestingly, rosmarinic acid was also completely accommodated in the catalytic site of glucosidase via mainly multiple H-bonds, a few hydrophobic bonds, and several van der Waals interactions (Figure 5D).

**Figure 6:**
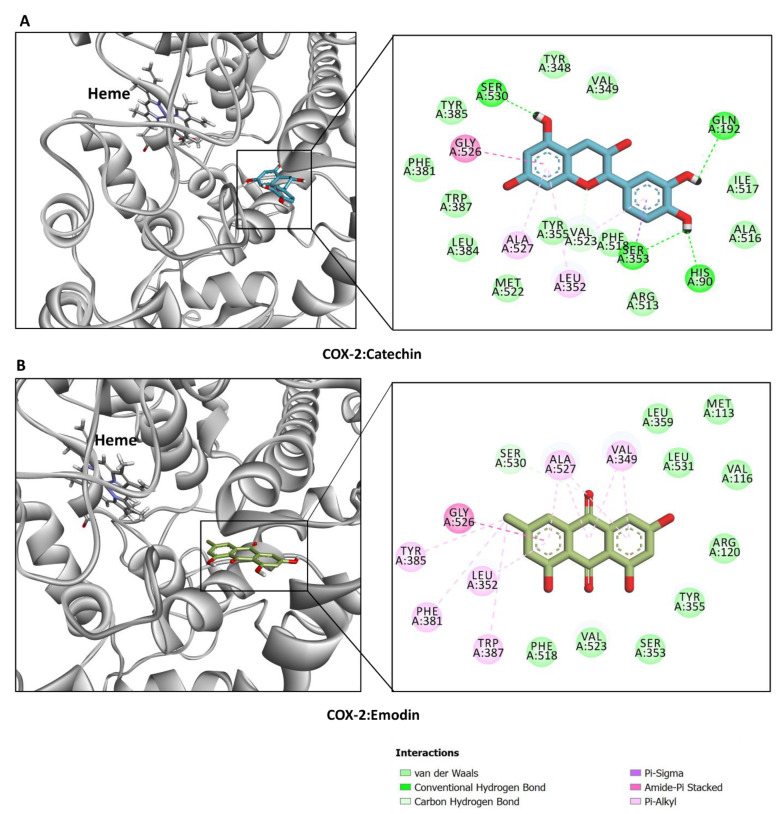
Binding mode and interaction of COX-2 crystal structure (PDB: 1CX2) with (**A**) catechin and (**B**) emodin.

Furthermore, to check the COX-2 inhibitory potential, these compounds were docked into the crystal structure of COX-2 [53]. Catechin and emodin were found to demonstrate the highest inhibitory potential against COX-2. While the dominant interactions in the case of catechin were H-bonds and hydrophobic interactions (Figure 6A), the binding of emodin is mainly driven by hydrophobic interactions deep inside the COX-2 active site, with van der Waals interactions reinforcing the binding (Figure 6B). Collectively, these findings suggest that the dominant compounds from different extracts from *N. italica* may have the potential to inhibit the biological activity of the target enzymes.

## 3. Materials and Methods

### 3.1. Plant Material

In the summer of 2021, we collected aerial parts of the tested *Nepeta italica* (Ovacik-Tunceli road, Tunceli) in Turkey. The plant specimens were identified by one of our co-authors, Dr. Ugur Cakilcioglu, and one specimen from the plants was deposited at the Munzur University herbarium. Prior to extraction, the plant materials were carefully washed with tap and distilled water to eliminate any soil and contaminants. After being air-dried for 10 days (in shade at room temperature), the aerial parts were powdered.

### 3.2. Preparation of Extracts

To obtain the extracts, we employed various solvents, including n-hexane, ethyl acetate, dichloromethane, ethanol, ethanol/water (70%), and water [54]. For the organic extracts, the maceration method was utilized. This involved mixing 10 g of plant material with 200 mL of each solvent and allowing it to rest at room temperature for 24 h. Subsequently, the mixtures were filtered through Whatman 1 filter paper, and the solvents were removed using a rotary evaporator. As for the water extracts, 10 g of plant material was infused in 200 milliliters of boiled water for 15 min, followed by filtration and lyophilization for 48 h. All extracts were then stored at 4 °C until analysis.

### 3.3. Determination of Total Bioactive Molecules

In order to determine the total content of bioactive compounds involved the determination of total phenols and flavonoids, the procedures described in paper [55] were used. Details are also reported in the Supplementary Material.

### 3.4. HPLC Determination of Phenolic Compounds

The extract was analyzed for phenol qualitative determination using a reversed-phase HPLC-DAD in gradient elution mode [56]. The details about separation and quantification are included in the Supplementary Material.

### 3.5. Biological Potential Assessment

The biological potential of the obtained extracts was determined by measuring their antioxidant and enzyme-inhibitory potential. The antioxidant potential was assessed using six in vitro tests (DPPH, ABTS, FRAP, CUPRAC, total antioxidant activity (PBD), and metal chelating (MCA) test). All used procedures are given in detail in the work of Nedić et al. [57]. The assessment of extracts’ inhibition of cholinesterase, tyrosinase, amylase, and glucosidase was performed in accordance with the protocol described in the study by Nedić, Nešović, Radišić, Gašić, Baošić, Joksimović, Pezo, Tešić, and Vovk [57]. The experiments were performed in triplicate, and differences between the extracts were compared using an ANOVA and Tukey’s test. Graph Pad Prism (version 9.2) was used for the analysis. Details about the above-mentioned assays are given in the Supplementary Material.

### 3.6. Ex Vivo Study

Isolated colon specimens were collected from euthanized mice [Project no. F4738.N.5QP authorized by the Italian Ministry of Health]. The tissue fragments were maintained at 5% CO_2_ at 37 °C for 4 h (incubation period) in RPMI buffer with added bacterial LPS (10 µg/mL) and in the presence of the extract (10–100 μg/mL). Details about the procedure are reported in the Supplementary Material.

RNA Extraction, Reverse Transcription, and Real-Time Reverse Transcription Polymerase Chain Reaction (RT-PCR)

Total RNA was extracted from colon specimens using TRI reagent (Sigma-Aldrich, St. Louis, MO, USA) according to the manufacturer’s protocol and reverse transcribed using a High-Capacity cDNA Reverse Transcription Kit (Thermo Fischer Scientific, Waltman, MA, USA). Details about the quantification of the gene expression of COX-2 and TNFα are included in the Supplementary Material. The relative quantification of the gene expression was carried out through the comparative 2^−ΔΔCt^ method [58]. Details about the gene expression analysis are given in the Supplementary Material.

### 3.7. Molecular Modeling

The prepared crystal protein structures of AChE (PDB ID: 6O52) [59], α-amylase (PDB ID: 1B2Y) [60], and BChE (PDB ID: 6EQP) [61] were retrieved from our previous studies [62,63]. Since crystal structures of human α-tyrosinase and α-glucosidase are not available, their homology models were built using the crystal structure of tyrosinase from *Priestia megaterium* (PDB ID: 6QXD) [64] and α-glucosidase from *Mus musculus* (PDB ID: 7KBJ) [65], respectively [66]. Furthermore, the crystal structure of COX-2 complexed with a selective inhibitor SC-558 (PDB: 1CX2) was retrieved from the protein data bank [53]. The 3D structures of all ligands were retrieved from the ChEMBL database (https://www.ebi.ac.uk/chembl/, accessed on 5 July 2023). The geometry of each ligand was optimized using UCSF Chimera [67]. MGLTools 1.5.6 software was used to merge all non-polar hydrogen atoms and add Gasteiger charges to all atoms. Docking was conducted using AutoDock 4.2.6 (https://autodock.scripts.edu, accessed on 5 July 2023) [68] in which an adopted docking protocol was applied [69]. Protein–ligand interaction was examined using the Biovia DS Visualizer v4.5 (BIOVIA, San Diego, CA, USA).

## 4. Conclusions

The present study explored the composition and biological properties of the aerial parts’ extracts from *N. italica*. Previous studies mostly explored the analgesic properties of the essential oil and antioxidant effects of the extracts, whereas the present work investigated different polarity extracts, with the determination of phenolic composition and scavenging/reducing and enzyme inhibition properties and the assessment of anti-inflammatory effects in an ex vivo experimental model of IBDs. All extracts were effective as antioxidants, enzyme inhibitors, and anti-inflammatory agents, and this was related, albeit partially, to the content of phenolic compounds. It is worthy to underline that polar extracts were more effective than lipophilic ones in blunting the LPS-induced increase of COX-2 and TNFα gene expression. This effect also agrees with the higher content in rosmarinic acid and its demonstrated efficacy as a protective agent in the inflamed colon, thus supporting future in vivo studies for confirming the efficacy of water and hydroalcoholic extracts of *N. italic* in IBDs, and a better definition of the limits of biocompatibility.

## Figures and Tables

**Table 1:** Total phenolic and flavonoid contents of the tested extracts.

Extracts	Total Phenolic Content (mg GAE/g)	Total Flavonoid Content (mg RE/g)
n-hexane	18.46 ± 0.23 ^e^	15.66 ± 0.46 ^c^
Dichloromethane	17.04 ± 0.16 ^e^	20.11 ± 0.37 ^a^
Ethyl acetate	21.10 ± 1.10 ^d^	17.45 ± 0.20 ^b^
Ethanol	28.41 ± 0.11 ^c^	15.28 ± 0.48 ^c^
Ethanol-Water	44.07 ± 0.91 ^b^	8.47 ± 0.12 ^e^
Water	59.13 ± 0.23 ^a^	11.46 ± 0.45 ^d^

Values are reported as mean ± SD of three parallel measurements. GAE: gallic acid equivalents; RE: rutin equivalents. Different letters indicate significant differences in the tested extracts (by ANOVA (Tukey’s test) assay, *p* < 0.05).

**Table 2:** Antioxidant properties of the tested extracts.

Extracts	DPPH (mg TE/g)	ABTS (mg TE/g)	CUPRAC (mg TE/g)	FRAP (mg TE/g)	PBD (mmol TE/g)	MCA (mg EDTAE/g)
n-hexane	3.88 ± 0.41 ^d^	10.54 ± 0.76 ^e^	37.03 ± 0.96 ^f^	19.33 ± 0.15 ^e^	2.14 ± 0.14 ^a^	56.60 ± 1.42 ^a^
Dichloromethane	5.11 ± 0.72 ^d^	19.79 ± 0.68 ^d^	48.63 ± 4.45 ^e^	21.35 ± 2.67 ^e^	2.13 ± 0.11 ^a^	43.62 ± 3.67 ^b^
Ethyl acetate	7.81 ± 0.78 ^c^	27.46 ± 2.28 ^c^	72.75 ± 1.98 ^d^	31.08 ± 1.08 ^d^	2.23 ± 0.01 ^a^	40.73 ± 3.96 ^b^
Ethanol	26.16 ± 0.05 ^b^	54.48 ± 0.21 ^b^	109.49 ± 1.64 ^c^	55.26 ± 0.97 ^c^	1.79 ± 0.02 ^b^	9.31 ± 0.75 ^d^
Ethanol-Water	49.25 ± 0.11 ^a^	89.35 ± 0.14 ^a^	184.29 ± 5.32 ^b^	95.64 ± 0.59 ^b^	1.24 ± 0.01 ^c^	23.13 ± 2.15 ^c^
Water	48.86 ± 0.13 ^a^	89.21 ± 0.05 ^a^	208.59 ± 1.72 ^a^	116.86 ± 1.47 ^a^	1.15 ± 0.04 ^c^	40.14 ± 1.65 ^b^

Values are reported as mean ± SD of three parallel measurements. PBD: phosphomolybdenum; MCA: metal chelating activity; TE: trolox equivalent; EDTAE: EDTA equivalent. Different letters indicate significant differences in the tested extracts (by ANOVA (Tukey’s test) assay, *p* < 0.05).

**Table 3:** Enzyme inhibitory effects of the tested extracts.

Extracts	AChE (mg GALAE/g)	BChE (mg GALAE/g)	Tyrosinase (mg KAE/g)	Amylase (mmol ACAE/g)	Glucosidase (mmol ACAE/g)
n-hexane	3.02 ± 0.47 ^a^	1.88 ± 0.20 ^bc^	72.12 ± 2.44 ^a^	0.37 ± 0.02 ^c^	4.91 ± 0.01 ^c^
Dichloromethane	2.93 ± 0.01 ^a^	2.40 ± 0.37 ^b^	49.91 ± 1.32 ^c^	0.58 ± 0.02 ^a^	0.14 ± 0.01 ^f^
Ethyl acetate	2.69 ± 0.17 ^a^	1.79 ± 0.40 ^bc^	56.29 ± 7.29 ^bc^	0.51 ± 0.01 ^b^	0.53 ± 0.07 ^e^
Ethanol	2.88 ± 0.03 ^a^	4.01 ± 0.28 ^a^	64.61 ± 0.94 ^ab^	0.33 ± 0.01 ^d^	5.38 ± 0.01 ^b^
Ethanol-Water	2.80 ± 0.02 ^a^	1.24 ± 0.07 ^c^	59.52 ± 1.31 ^b^	0.25 ± 0.01 ^e^	5.60 ± 0.01 ^a^
Water	0.04 ± 0.01 ^b^	na	15.04 ± 0.22 ^d^	0.05 ± 0.01 ^f^	0.94 ± 0.04 ^d^

Values are reported as mean ± SD of three parallel measurements. GALAE: galantamine equivalent; KAE: kojic acid equivalent; ACAE: acarbose equivalent; na: not active. Different letters indicate significant differences in the tested extracts (by ANOVA (Tukey’s test) assay, *p* < 0.05).

## Data Availability

The data that support the findings of this study are available from the corresponding author.

## Supplementary Materials

The following supporting information can be downloaded at: https://www.mdpi.com/article/10.3390/plants12152785/s1. Tables S1 and S2, Figures S1 and S2. Reference [70] is cited in the supplementary materials.

## References

[1] Salehi B., Zucca P., Sharifi-Rad M., Pezzani R., Rajabi S., Setzer W.N., Varoni E.M., Iriti M., Kobarfard F., Sharifi-Rad J. (2018). Phytotherapeutics in cancer invasion and metastasis. Phytother. Res..

[2] Formisano C., Rigano D., Senatore F. (2011). Chemical constituents and biological activities of Nepeta species. Chem. Biodivers..

[3] Süntar I., Nabavi S.M., Barreca D., Fischer N., Efferth T. (2018). Pharmacological and chemical features of Nepeta L. genus: Its importance as a therapeutic agent. Phytother. Res..

[4] Aydin S., Demir T., Öztürk Y., Başer K.H.C. (1999). Analgesic activity of *Nepeta italica* L. Phytother. Res. Int. J. Devoted Pharmacol. Toxicol. Eval. Nat. Prod. Deriv..

[5] Kaska A., Deniz N., Çiçek M., Mammadov R. (2018). Evaluation of Antioxidant Properties, Phenolic Compounds, Anthelmintic, and Cytotoxic Activities of Various Extracts Isolated from Nepeta cadmea: An Endemic Plant for Turkey. J. Food Sci..

[6] Goldansaz S.M., Festa C., Pagano E., De Marino S., Finamore C., Parisi O.A., Borrelli F., Sonboli A., D’Auria M.V. (2019). Phytochemical and biological studies of *Nepeta asterotricha* Rech. f.(Lamiaceae): Isolation of nepetamoside. Molecules.

[7] Strober W., Fuss I., Mannon P. (2007). The fundamental basis of inflammatory bowel disease. J. Clin. Investig..

[8] Choi C.H., Moon W., Kim Y.S., Kim E.S., Lee B.-I., Jung Y., Yoon Y.S., Lee H., Park D.I., Han D.S. (2017). Second Korean guidelines for the management of ulcerative colitis. Intest. Res..

[9] Ferrante C., Recinella L., Ronci M., Orlando G., Di Simone S., Brunetti L., Chiavaroli A., Leone S., Politi M., Tirillini B. (2019). Protective effects induced by alcoholic Phlomis fruticosa and Phlomis herba-venti extracts in isolated rat colon: Focus on antioxidant, anti-inflammatory, and antimicrobial activities in vitro. Phytother. Res..

[10] Kainama H., Fatmawati S., Santoso M., Papilaya P.M., Ersam T. (2020). The relationship of free radical scavenging and total phenolic and flavonoid contents of Garcinia lasoar PAM. Pharm. Chem. J..

[11] Kitzberger C.S.G., Smânia Jr A., Pedrosa R.C., Ferreira S.R.S. (2007). Antioxidant and antimicrobial activities of shiitake (Lentinula edodes) extracts obtained by organic solvents and supercritical fluids. J. Food Eng..

[12] An L., Wang G., Jia H., Liu C., Sui W., Si C. (2017). Fractionation of enzymatic hydrolysis lignin by sequential extraction for enhancing antioxidant performance. Int. J. Biol. Macromol..

[13] Dirar A., Alsaadi D., Wada M., Mohamed M., Watanabe T., Devkota H. (2019). Effects of extraction solvents on total phenolic and flavonoid contents and biological activities of extracts from Sudanese medicinal plants. S. Afr. J. Bot..

[14] Galanakis C., Goulas V., Tsakona S., Manganaris G., Gekas V. (2013). A knowledge base for the recovery of natural phenols with different solvents. Int. J. Food Prop..

[15] Švarc-Gajić J., Stojanović Z., Carretero A.S., Román D.A., Borrás I., Vasiljević I. (2013). Development of a microwave-assisted extraction for the analysis of phenolic compounds from Rosmarinus officinalis. J. Food Eng..

[16] Kaska A., Çiçek M., Mammadov R. (2019). Biological activities, phenolic constituents and mineral element analysis of two endemic medicinal plants from Turkey: Nepeta italica subsp. cadmea and Teucrium sandrasicum. S. Afr. J. Bot..

[17] Manukyan A. (2013). Effects of PAR and UV-B radiation on herbal yield, bioactive compounds and their antioxidant capacity of some medicinal plants under controlled environmental conditions. Photochem. Photobiol..

[18] Al Jitan S., Alkhoori S.A., Yousef L.F. (2018). Phenolic acids from plants: Extraction and application to human health. Stud. Nat. Prod. Chem..

[19] Sharma A., Cooper R., Bhardwaj G., Cannoo D.S. (2021). The genus Nepeta: Traditional uses, phytochemicals and pharmacological properties. J. Ethnopharmacol..

[20] Sun W., Shahrajabian M.H. (2023). Therapeutic potential of phenolic compounds in medicinal plants—Natural health products for human health. Molecules.

[21] Wannes W.A., Mhamdi B., Sriti J., Jemia M.B., Ouchikh O., Hamdaoui G., Kchouk M.E., Marzouk B. (2010). Antioxidant activities of the essential oils and methanol extracts from myrtle (*Myrtus communis* var. italica L.) leaf, stem and flower. Food Chem. Toxicol..

[22] Bubalo M.C., Ćurko N., Tomašević M., Ganić K.K., Redovniković I.R. (2016). Green extraction of grape skin phenolics by using deep eutectic solvents. Food Chem..

[23] Flórez N., Conde E., Domínguez H. (2015). Microwave assisted water extraction of plant compounds. J. Chem. Technol. Biotechnol..

[24] Muhamad I.I., Hassan N.D., Mamat S.N., Nawi N.M., Rashid W.A., Tan N.A. (2017). Extraction technologies and solvents of phytocompounds from plant materials: Physicochemical characterization and identification of ingredients and bioactive compounds from plant extract using various instrumentations. Ingredients Extraction by Physicochemical Methods in Food.

[25] Ribeiro T.B., Oliveira A., Campos D., Nunes J., Vicente A.A., Pintado M. (2020). Simulated digestion of an olive pomace water-soluble ingredient: Relationship between the bioaccessibility of compounds and their potential health benefits. Food Funct..

[26] Boulaaba M., Kalai F.Z., Dakhlaoui S., Ezzine Y., Selmi S., Bourgou S., Smaoui A., Isoda H., Ksouri R. (2019). Antioxidant, antiproliferative and anti-inflammatory effects of Glaucium flavum fractions enriched in phenolic compounds. Med. Chem. Res..

[27] Kassim M., Achoui M., Mustafa M.R., Mohd M.A., Yusoff K.M. (2010). Ellagic acid, phenolic acids, and flavonoids in Malaysian honey extracts demonstrate in vitro anti-inflammatory activity. Nutr. Res..

[28] Choi C.W., Kim S.C., Hwang S.S., Choi B.K., Ahn H.J., Lee M.Y., Park S.H., Kim S.K. (2002). Antioxidant activity and free radical scavenging capacity between Korean medicinal plants and flavonoids by assay-guided comparison. Plant Sci..

[29] Rebaya A., Belghith S.I., Baghdikian B., Leddet V.M., Mabrouki F., Olivier E., kalthoum Cherif J., Ayadi M.T. (2015). Total phenolic, total flavonoid, tannin content, and antioxidant capacity of Halimium halimifolium (Cistaceae). J. Appl. Pharm. Sci..

[30] Albayrak S., Aksoy A. (2023). Comparative analysis of phenolic compositions and biological activities of three endemic Teucrium L.(Lamiaceae) species from Turkey. An. Acad. Bras. Ciências.

[31] Jafri L., Saleem S., Ullah N., Mirza B. (2017). In vitro assessment of antioxidant potential and determination of polyphenolic compounds of Hedera nepalensis K. Koch. Arab. J. Chem..

[32] Lou S.-N., Hsu Y.-S., Ho C.-T. (2014). Flavonoid compositions and antioxidant activity of calamondin extracts prepared using different solvents. J. Food Drug Anal..

[33] Khan T., Ipshita A., Mazumdar R., Abdullah A., Islam G., Rahman M. (2020). Bioactive polyphenol profiling and in-vitro antioxidant activity of Tinospora cordifolia Miers ex Hook F and Thoms: A potential ingredient for functional food development. Bangladesh J. Sci. Ind. Res..

[34] Nirmala C., Bisht M.S., Bajwa H.K., Santosh O. (2018). Bamboo: A rich source of natural antioxidants and its applications in the food and pharmaceutical industry. Trends Food Sci. Technol..

[35] Gong L., Feng D., Wang T., Ren Y., Liu Y., Wang J. (2020). Inhibitors of α-amylase and α-glucosidase: Potential linkage for whole cereal foods on prevention of hyperglycemia. Food Sci. Nutr..

[36] Thilagam E., Parimaladevi B., Kumarappan C., Mandal S.C. (2013). α-Glucosidase and α-amylase inhibitory activity of Senna surattensis. J. Acupunct. Meridian Stud..

[37] Joshi M., Kumar R., Prakash O., Pant A.K., Rawat D. (2021). Chemical composition and biological activities of Nepeta hindostana (Roth) Haines, Nepeta graciliflora Benth. and Nepeta cataria L. from India. J. Med. Herbs.

[38] Salehi B., Valussi M., Jugran A.K., Martorell M., Ramírez-Alarcón K., Stojanović-Radić Z.Z., Antolak H., Kręgiel D., Mileski K.S., Sharifi-Rad M. (2018). Nepeta species: From farm to food applications and phytotherapy. Trends Food Sci. Technol..

[39] Zengin G., Mahomoodally M.F., Aktumsek A., Jekő J., Cziáky Z., Rodrigues M.J., Custodio L., Polat R., Cakilcioglu U., Ayna A. (2021). Chemical profiling and biological evaluation of Nepeta baytopii extracts and essential oil: An endemic plant from Turkey. Plants.

[40] Nordberg A., Ballard C., Bullock R., Darreh-Shori T., Somogyi M. (2013). A review of butyrylcholinesterase as a therapeutic target in the treatment of Alzheimer’s disease. Prim. Care Companion CNS Disord..

[41] Norel X., Angrisani M., Labat C., Gorenne I., Dulmet E., Rossi F., Brink C. (1993). Degradation of acetylcholine in human airways: Role of butyrylcholinesterase. Br. J. Pharmacol..

[42] Yilmaz A., Boga M., Topcu G. (2016). Novel terpenoids with potential anti-alzheimer activity from Nepeta obtusicrena. Rec. Nat. Prod..

[43] Yilmaz A., Cağlar P., Dirmenci T., Gören N., Topcu G. (2012). A novel isopimarane diterpenoid with acetylcholinesterase inhibitory activity from Nepeta sorgerae, an endemic species to the Nemrut Mountain. Nat. Prod. Commun..

[44] Lai X., Wichers H.J., Soler-Lopez M., Dijkstra B.W. (2018). Structure and function of human tyrosinase and tyrosinase-related proteins. Chem. —A Eur. J..

[45] Tief K., Hahne M., Schmidt A., Beermann F. (1996). Tyrosinase, the key enzyme in melanin synthesis, is expressed in murine brain. Eur. J. Biochem..

[46] Achitei D., Ciobica A., Balan G., Gologan E., Stanciu C., Stefanescu G. (2013). Different profile of peripheral antioxidant enzymes and lipid peroxidation in active and non-active inflammatory bowel disease patients. Dig. Dis. Sci..

[47] Koutroubakis I.E., Malliaraki N., Dimoulios P.D., Karmiris K., Castanas E., Kouroumalis E.A. (2004). Decreased total and corrected antioxidant capacity in patients with inflammatory bowel disease. Dig. Dis. Sci..

[48] Rezaie A., Parker R.D., Abdollahi M. (2007). Oxidative stress and pathogenesis of inflammatory bowel disease: An epiphenomenon or the cause?. Dig. Dis. Sci..

[49] Chung H.-L., Yue G.G.-L., To K.-F., Su Y.-L., Huang Y., Ko W.-H. (2007). Effect of Scutellariae Radix extract on experimental dextran-sulfate sodium-induced colitis in rats. World J. Gastroenterol. WJG.

[50] Lenoir L., Joubert-Zakeyh J., Texier O., Lamaison J.L., Vasson M.P., Felgines C. (2012). Aloysia triphylla infusion protects rats against dextran sulfate sodium-induced colonic damage. J. Sci. Food Agric..

[51] Recinella L., Chiavaroli A., Ronci M., Menghini L., Brunetti L., Leone S., Tirillini B., Angelini P., Covino S., Venanzoni R. (2020). Multidirectional Pharma-Toxicological Study on *Harpagophytum procumbens* DC. ex Meisn.: An IBD-Focused Investigation. Antioxidants.

[52] Recinella L., Chiavaroli A., Veschi S., Cama A., Acquaviva A., Libero M.L., Leone S., Di Simone S.C., Pagano E., Zengin G. (2022). A grape (*Vitis vinifera* L.) pomace water extract modulates inflammatory and immune response in SW-480 cells and isolated mouse colon. Phytother. Res..

[53] Kurumbail R.G., Stevens A.M., Gierse J.K., McDonald J.J., Stegeman R.A., Pak J.Y., Gildehaus D., Iyashiro J.M., Penning T.D., Seibert K. (1996). Structural basis for selective inhibition of cyclooxygenase-2 by anti-inflammatory agents. Nature.

[54] Acquaviva A., Nilofar N., Bouyahya A., Zengin G., Di Simone S.C., Recinella L., Leone S., Brunetti L., Uba A.I., Cakilcioğlu U. (2023). Chemical Characterization of Different Extracts from Artemisia annua and Their Antioxidant, Enzyme Inhibitory and Anti-Inflammatory Properties. Chem. Biodivers..

[55] Uysal S., Zengin G., Locatelli M., Bahadori M.B., Mocan A., Bellagamba G., De Luca E., Mollica A., Aktumsek A. (2017). Cytotoxic and enzyme inhibitory potential of two Potentilla species (P. speciosa L. and P. reptans Willd.) and their chemical composition. Front. Pharmacol..

[56] di Giacomo V., Recinella L., Chiavaroli A., Orlando G., Cataldi A., Rapino M., Di Valerio V., Politi M., Antolini M.D., Acquaviva A. (2021). Metabolomic Profile and Antioxidant/Anti-Inflammatory Effects of Industrial Hemp Water Extract in Fibroblasts, Keratinocytes and Isolated Mouse Skin Specimens. Antioxidants.

[57] Nedić N., Nešović M., Radišić P., Gašić U., Baošić R., Joksimović K., Pezo L., Tešić Ž., Vovk I. (2022). Polyphenolic and Chemical Profiles of Honey From the Tara Mountain in Serbia. Front. Nutr..

[58] Livak K.J., Schmittgen T.D. (2001). Analysis of relative gene expression data using real-time quantitative PCR and the 2−ΔΔCT method. Methods.

[59] Gerlits O., Ho K.-Y., Cheng X., Blumenthal D., Taylor P., Kovalevsky A., Radić Z. (2019). A new crystal form of human acetylcholinesterase for exploratory room-temperature crystallography studies. Chem. Biol. Interact..

[60] Maurus R., Begum A., Williams L.K., Fredriksen J.R., Zhang R., Withers S.G., Brayer G.D. (2008). Alternative Catalytic Anions Differentially Modulate Human α-Amylase Activity and Specificity. Biochemistry.

[61] Rosenberry T., Brazzolotto X., Macdonald I., Wandhammer M., Trovaslet-Leroy M., Darvesh S., Nachon F. (2017). Comparison of the Binding of Reversible Inhibitors to Human Butyrylcholinesterase and Acetylcholinesterase: A Crystallographic, Kinetic and Calorimetric Study. Molecules.

[62] Fahmy N.M., Fayez S., Uba A.I., Shariati M.A., Aljohani A.S.M., El-Ashmawy I.M., Batiha G.E.-S., Eldahshan O.A., Singab A.N., Zengin G. (2023). Comparative GC-MS Analysis of Fresh and Dried Curcuma Essential Oils with Insights into Their Antioxidant and Enzyme Inhibitory Activities. Plants.

[63] Eltayeb L.M.H., Yagi S., Mohamed H.M.M., Zengin G., Shariati M.A., Rebezov M., Uba A.I., Lorenzo J.M. (2023). Essential Oils Composition and Biological Activity of Chamaecyparis obtusa, Chrysopogon nigritanus and Lavandula coronopifolia Grown Wild in Sudan. Molecules.

[64] Ielo L., Deri B., Germanò M.P., Vittorio S., Mirabile S., Gitto R., Rapisarda A., Ronsisvalle S., Floris S., Pazy Y. (2019). Exploiting the 1-(4-fluorobenzyl)piperazine fragment for the development of novel tyrosinase inhibitors as anti-melanogenic agents: Design, synthesis, structural insights and biological profile. Eur. J. Med. Chem..

[65] Karade S.S., Hill M.L., Kiappes J.L., Manne R., Aakula B., Zitzmann N., Warfield K.L., Treston A.M., Mariuzza R.A. (2021). N-Substituted Valiolamine Derivatives as Potent Inhibitors of Endoplasmic Reticulum α-Glucosidases I and II with Antiviral Activity. J. Med. Chem..

[66] Chiavaroli A., Libero M.L., Di Simone S.C., Acquaviva A., Nilofar, Recinella L., Leone S., Brunetti L., Cicia D., Izzo A.A. (2023). Adding New Scientific Evidences on the Pharmaceutical Properties of Pelargonium quercetorum Agnew Extracts by Using In Vitro and In Silico Approaches. Plants.

[67] Pettersen E.F., Goddard T.D., Huang C.C., Couch G.S., Greenblatt D.M., Meng E.C., Ferrin T.E. (2004). UCSF Chimera?A visualization system for exploratory research and analysis. J. Comput. Chem..

[68] Morris G.M., Huey R., Lindstrom W., Sanner M.F., Belew R.K., Goodsell D.S., Olson A.J. (2009). AutoDock4 and AutoDockTools4: Automated docking with selective receptor flexibility. J. Comput. Chem..

[69] Llorent-Martínez E.J., Ruiz-Medina A., Zengin G., Ak G., Jugreet S., Mahomoodally M.F., Emre G., Orlando G., Libero M.L., Nilofar (2022). New Biological and Chemical Evidences of Two Lamiaceae Species (Thymbra capitata and Thymus sipyleus subsp. rosulans): In Vitro, In Silico and Ex Vivo Approaches. Molecules.

[70] El-Naggar E.M.B., Azazi M., Švajdlenka E. (2013). Artemisinin from minor to major ingredient in *Artemisia annua* cultivated in Egypt. J. Appl. Pharm. Sci..

