# Supplementary material for: Screening for Chemical Characterization and Pharmacological Properties of Different Extracts from Nepeta italica
Source: Plants (Basel). 2023 Jul 27;12(15):2785. doi: 10.3390/plants12152785 (PMC10420686; doi:10.3390/plants12152785)
Supplement: Supplementary file 1 [file plants-12-02785-s001.zip › plants-2519537-supplementary.pdf]

**Assays for Total Phenolic and Flavonoid Contents**

The total phenolic content was determined by employing the methods given in the literature with some modification. Sample solution (0.25 mL) was mixed with diluted Folin–Ciocalteu reagent (1 mL, 1:9, v/v) and shaken vigorously. After 3 min, $Na_2CO_3$ solution (0.75 mL, 1%) was added and the sample absorbance was read at 760 nm after a 2 h incubation at room temperature. The total phenolic content was expressed as milligrams of gallic acid equivalents (mg GAE/g extract)(Uysal et al., 2017). All standards were purchased from Merck Life Science S.r.l. (Milan, Italy), and have a purity ≥95%.

The total flavonoid content was determined using the $AlCl_3$ method. Briefly, sample solution (1 mL) was mixed with the same volume of aluminum trichloride (2%) in methanol. Similarly, a blank was prepared by adding sample solution (1 mL) to methanol (1 mL) without $AlCl_3$. The sample and blank absorbances were read at 415 nm after a 10 min incubation at room temperature. The absorbance of the blank was subtracted from that of the sample. Rutin was used as a reference standard and the total flavonoid content was expressed as milligrams of rutin equivalents (mg RE/g extract) (Uysal et al., 2017).

**Determination of Antioxidant and Enzyme Inhibitory Effects**

Antioxidant (DPPH and ABTS radical scavenging, reducing power (CUPRAC and FRAP), phosphomolybdenum and metal chelating (ferrozine method)) and enzyme inhibitory activities (cholinesterase (Elmann's method), tyrosinase (dopachrome method), $\alpha$-amylase (iodine/potassium iodide method), $\alpha$ -glucosidase (chromogenic PNPG method) and pancreatic lipase (*p*-nitrophenyl butyrate (p-NPB) method) were determined using the methods previously described by Uysal et al. (Uysal et al., 2017) and Grochowski et al. (Grochowski et al., 2017). All standards were purchased from Merck Life Science S.r.l. (Milan, Italy), and have a purity ≥95%.

For the DPPH (1,1-diphenyl-2-picrylhydrazyl) radical scavenging assay: Sample solution was added to 4 mL of a 0.004% methanol solution of DPPH. The sample absorbance was read at 517 nm after a 30 min incubation at room temperature in the dark. DPPH radical scavenging activity was expressed as milligrams of trolox equivalents (mg TE/g extract).

For ABTS (2,2′-azino-bis(3-ethylbenzothiazoline) 6-sulfonic acid) radical scavenging assay: Briefly, ABTS+ was produced directly by reacting 7 mM ABTS solution with 2.45 mM potassium persulfate and allowing the mixture to stand for 12–16 in the dark at room temperature. Prior to beginning the assay, ABTS solution was diluted with methanol to an absorbance of 0.700 ± 0.02 at 734 nm. Sample solution was added to ABTS solution (2 mL) and mixed. The sample absorbance was read at 734 nm after a 30 min incubation at room temperature. The ABTS radical scavenging activity was expressed as milligrams of trolox equivalents (mg TE/g extract).

For CUPRAC (cupric ion reducing activity) activity assay: Sample solution was added to premixed reaction mixture containing $CuCl_2$ (1 mL, 10 mM), neocuproine (1 mL, 7.5 mM) and $NH_4Ac$ buffer (1 mL, 1 M, pH 7.0). Similarly, a blank was prepared by adding sample solution (0.5 mL) to premixed reaction mixture (3 mL) without $CuCl_2$ . Then, the sample and blank absorbances were read at 450 nm after a 30 min incubation at room temperature. The absorbance of the blank was subtracted from that of the sample. CUPRAC activity was expressed as milligrams of trolox equivalents (mg TE/g extract).

For FRAP (ferric reducing antioxidant power) activity assay: Sample solution was added to premixed FRAP reagent (2 mL) containing acetate buffer (0.3 M, pH 3.6), 2,4,6-tris(2-pyridyl)-S-triazine (TPTZ) (10 mM) in 40 mM HCl and ferric chloride (20 mM) in a ratio of 10:1:1 (v/v/v). Then, the sample absorbance was read at 593 nm after a 30 min incubation at room temperature. FRAP activity was expressed as milligrams of trolox equivalents (mg TE/g extract).

For phosphomolybdenum method: Sample solution was combined with 3 mL of reagent solution (0.6 M sulfuric acid, 28 mM sodium phosphate and 4 mM ammonium molybdate). The sample absorbance was read at 695 nm after a 90 min incubation at 95 °C. The total antioxidant capacity was expressed as millimoles of trolox equivalents (mmol TE/g extract).

For metal chelating activity assay: Briefly, sample solution was added to $FeCl_2$ solution (0.05 mL, 2 mM). The reaction was initiated by the addition of 5 mM ferrozine (0.2 mL). Similarly, a blank was prepared by adding sample solution (2 mL) to $FeCl_2$ solution (0.05 mL, 2 mM) and water (0.2 mL) without ferrozine. Then, the sample and blank absorbances were read at 562 nm after 10 min incubation at room temperature. The absorbance of the blank was sub-tracted from that of the sample. The metal chelating activity was expressed as milligrams of EDTA (disodium edetate) equivalents (mg EDTAE/g extract).

For Cholinesterase (ChE) inhibitory activity assay: Sample solution (was mixed with DTNB (5,5-dithio-bis(2-nitrobenzoic) acid, Sigma, St. Louis, MO, USA) (125 μL) and AChE (acetylcholines-terase (Electric ell acetylcholinesterase, Type-VI-S, EC 3.1.1.7,Sigma)), or BChE (butyrylcholinesterase (horse serum butyrylcholinesterase, EC 3.1.1.8, Sigma)) solution (25 μL) in Tris–HCl buffer (pH 8.0) in a 96-well microplate and incubated for 15 min at 25 °C. The reaction was then initiated with the addition of acetylthiocholine iodide (ATCI, Sigma) or butyrylthiocholine chloride (BTCl, Sigma) (25 μL). Similarly, a blank was prepared by adding sample solution to all reaction reagents without enzyme (AChE or BChE) solution. The sample and blank absorbances were read at 405 nm after 10 min incubation at 25 °C. The absorbance of the blank was subtracted from that of the sample and the cholinesterase inhibitory activity was expressed as galanthamine equivalents (mgGALAE/g extract).

For Tyrosinase inhibitory activity assay: Sample solution was mixed with tyrosinase solution (40 μL, Sigma) and phosphate buffer (100 μL, pH 6.8) in a 96-well microplate and incubated for 15 min at 25 °C. The reaction was then initiated with the addition of L-DOPA (40 μL, Sigma). Similarly, a blank was prepared by adding sample solution to all reaction reagents without enzyme (tyrosinase) solution. The sample and blank absorbances were read at 492 nm after a 10 min incubation at 25 °C. The absorbance of the blank was subtracted from that of the sample and the tyrosinase inhibitory activity was expressed as kojic acid equivalents (mgKAE/g extract).

For $\alpha$-amylase inhibitory activity assay: Sample solution was mixed with $\alpha$-amylase solution (ex-porcine pancreas, EC 3.2.1.1, Sigma) (50 μL) in phosphate buffer (pH 6.9 with 6 mM sodium chloride) in a 96-well microplate and incubated for 10 min at 37 °C. After pre-incubation, the reaction was initiated with the addition of starch solution (50 μL, 0.05%). Similarly, a blank was prepared by adding

sample solution to all reaction reagents without enzyme ($\alpha$-amylase) solution. The reaction mixture was incubated 10 min at 37 °C. The reaction was then stopped with the addition of HCl (25 μL, 1 M). This was followed by addition of the iodine-potassium iodide solution (100 μL). The sample and blank absorbances were read at 630 nm. The absorbance of the blank was subtracted from that of the sample and the $\alpha$-amylase inhibitory activity was expressed as acarbose equivalents (mmol ACE/g extract).

For $\alpha$-glucosidase inhibitory activity assay: Sample solution was mixed with glutathione (50 μL), $\alpha$-glucosidase solution (from Saccharomyces cerevisiae, EC 3.2.1.20, Sigma) (50 μL) in phosphate buffer (pH 6.8) and PNPG (4-N-trophenyl-$\alpha$-D-glucopyranoside, Sigma) (50 μL) in a 96-well microplate and incubated for 15 min at 37 °C. Similarly, a blank was prepared by adding sample solution to all reaction reagents without enzyme ($\alpha$-glucosidase) solution. The reaction was then stopped with the addition of sodium carbonate (50 μL, 0.2 M). The sample and blank absorbances were read at 400 nm. The absorbance of the blank was subtracted from that of the sample and the $\alpha$-glucosidase inhibitory activity was expressed as acarbose equivalents (mmol ACE/g extract).

Grochowski, D. M., Uysal, S., Aktumsek, A., Granica, S., Zengin, G., Ceylan, R., . . . Tomczyk, M. (2017). In vitro enzyme inhibitory properties, antioxidant activities, and phytochemical profile of Potentilla thuringiaca. Phytochemistry Letters, 20, 365-372.

Uysal, S., Zengin, G., Locatelli, M., Bahadori, M. B., Mocan, A., Bellagamba, G., . . . Aktumsek, A. (2017). Cytotoxic and enzyme inhibitory potential of two Potentilla species (P. speciosa L. and P. reptans Willd.) and their chemical composition. Frontiers in pharmacology, 8, 290.

## HPLC Determination of Phenolic Compounds

The HPLC apparatus consisted of two PU-2080 PLUS chromatographic pumps, a DG-2080-54 line degasser, a mix-2080-32 mixer, diode array (DAD) detector, and a CO-2060 PLUS column thermostat (all from Jasco, Tokyo, Japan). Integration was performed by ChromNAV2 Chromatography software. Before the injection in the HPLC apparatus, the extract was centrifuged at 3500×g for 15 min, and the supernatant was diluted to 20 mg/ml.

The extract was analyzed for phenol qualitative determination using a reversed-phase HPLC-DAD in gradient elution mode (Chiavaroli et al., 2023). The separation was conducted within 60 min of the chromatographic run, starting from the following separation conditions: 97 % water with 0.1% formic acid, 3% methanol with 0.1% formic acid (Table S1). The separation was performed on an Infinity lab Poroshell 120-SB reverse phase column (C18, 150 × 4.6 mm i.d., 2.7 μm; Agilent, Santa Clara, CA, USA). Column temperature was set at 30 °C. All standards were purchased from Merck Life Science S.r.l. (Milan, Italy), and have a purity ≥95%. The area under the curve from HPLC chromatograms was used to carry out a semiquantitative determination of the analyte concentrations in the extract (Chiavaroli et al., 2023).

**Table S1**. Gradient elution condition of the HPLC-DAD-MS analyses.

| TIME (min) | COMPOSITION A% (Water + Formic acid 0.1%) | COMPOSITION B% (Methanol + Formic acid 0.1%) | FLOW (mL/min) |
|---|---|---|---|
| 1 | 97 | 3 | 0.6 |
| 5 | 77 | 23 | 0.6 |
| 12 | 73 | 27 | 0.6 |
| 18 | 57 | 43 | 0.6 |
| 25 | 52 | 48 | 0.6 |
| 32 | 50 | 50 | 0.6 |
| 34 | 50 | 50 | 0.6 |
| 37 | 35 | 65 | 0.6 |
| 40 | 5 | 95 | 0.6 |
| 47 | 10 | 90 | 0.6 |
| 48 | 10 | 90 | 0.6 |

**Table S2.** Retention times and peak area of the content in specialized metabolites of the tested *Nepeta italica* extracts. All identified phytochemicals have been identified through comparison with pure standards. Determination of the compounds was performed via DAD detector at 232–372 nm wavelength; nd: not detected.

| Components | | Rt (min) | Hexane | DCM | EA | EtOH | EtOH/Water | Water |
|---|---|---|---|---|---|---|---|---|
| **1** | Gallic acid | 8.80 | nd | nd | nd | 4779 | 27517 | 29929 |
| **2** | 3-Hydroxytyrosol | 11.71 | 1494 | 183 | 483 | 9352 | 16864 | 15334 |
| **3** | Caftaric acid | 12.93 | nd | 4979 | 142 | nd | 8389 | 6354 |
| **4** | Catechin | 14.80 | nd | nd | 84 | 2016 | 54161 | nd |
| **5** | Gentisic acid | 15.82 | nd | nd | nd | 2453 | nd | nd |
| **6** | 4-Hydroxybenzoic acid | 16.20 | nd | 1698 | 8227 | 6348 | 5168 | 6925 |
| **7** | Loganic acid | 16.60 | 1280 | nd | 182 | 1184 | 1298 | 17522 |
| **8** | Chlorogenic acid | 16.81 | nd | nd | 509 | 15816 | 29636 | nd |
| **9** | Vanillic acid | 18.60 | 4149 | 1724 | 1678 | 30270 | 30151 | 23517 |
| **10** | Caffeic acid | 19.00 | 779 | 400 | 3203 | 12238 | 18885 | 18347 |
| **11** | Epicatechin | 19.41 | 1201 | 2381 | 2261 | 5986 | 2440 | 2972 |
| **12** | Syringic acid | 20.05 | nd | 544 | 2312 | nd | 4268 | 4727 |
| **13** | Syringaldehyde | 21.80 | nd | 333 | 269 | 2184 | 3454 | 4814 |
| **14** | p-Coumaric acid | 23.06 | 455 | 822 | 1451 | 2430 | 7781 | 7661 |
| **15** | t-Ferulic acid | 24.00 | 778 | 366 | 673 | 1762 | 3897 | 6227 |
| **16** | Benzoic acid | 26.38 | 688 | 4978 | 4668 | 17728 | 34340 | 15472 |
| **17** | Hyperoside | 26.92 | 10931 | 2449 | 1898 | nd | 5950 | nd |
| **18** | Rutin | 27.16 | nd | nd | nd | 32643 | 12090 | 7943 |
| **19** | Resveratrol | 27.70 | 3704 | nd | 2327 | 6335 | 4923 | 2811 |
| **20** | Rosmarinic acid | 28.53 | 757 | 972 | 13187 | 378613 | 493307 | 345699 |
| **21** | t-Cinnamic acid | 34.39 | 1287 | 4457 | 9333 | 28733 | 39181 | 13455 |
| **22** | Quercetin | 35.89 | 4423 | nd | 1386 | nd | nd | 3190 |
| **23** | Naringenin | 36.77 | nd | nd | 2412 | nd | nd | nd |
| **24** | 2.3-Dimethylbenzoic acid | 37.77 | nd | 3499 | 2802 | nd | nd | nd |
| **25** | Hesperetin | 39.38 | nd | 6282 | 4195 | 19402 | nd | nd |

| | | | | | | | |
|---|---|---|---|---|---|---|---|
| **26** | Kaempferol | 41.74 | 2238 | 2678 | 6566 | 704 | 26981 | nd |
| **27** | Carvacrol | 44.69 | 50677 | 42365 | 58403 | 52718 | 11689 | 2974 |
| **28** | Thymol | 44.92 | 315 | 61193 | 48274 | 49520 | 16730 | 20986 |
| **29** | Flavone | 45.60 | 334637 | 27156 | 51808 | 32370 | 5843 | 2092 |
| **30** | 3-Hydroxyflavone | 46.05 | 20111 | 299086 | 249749 | 194952 | 32460 | 14411 |
| **31** | Emodin | 47.70 | 6970 | 135273 | 101522 | 65198 | nd | 8257 |

**Hexane (A)**

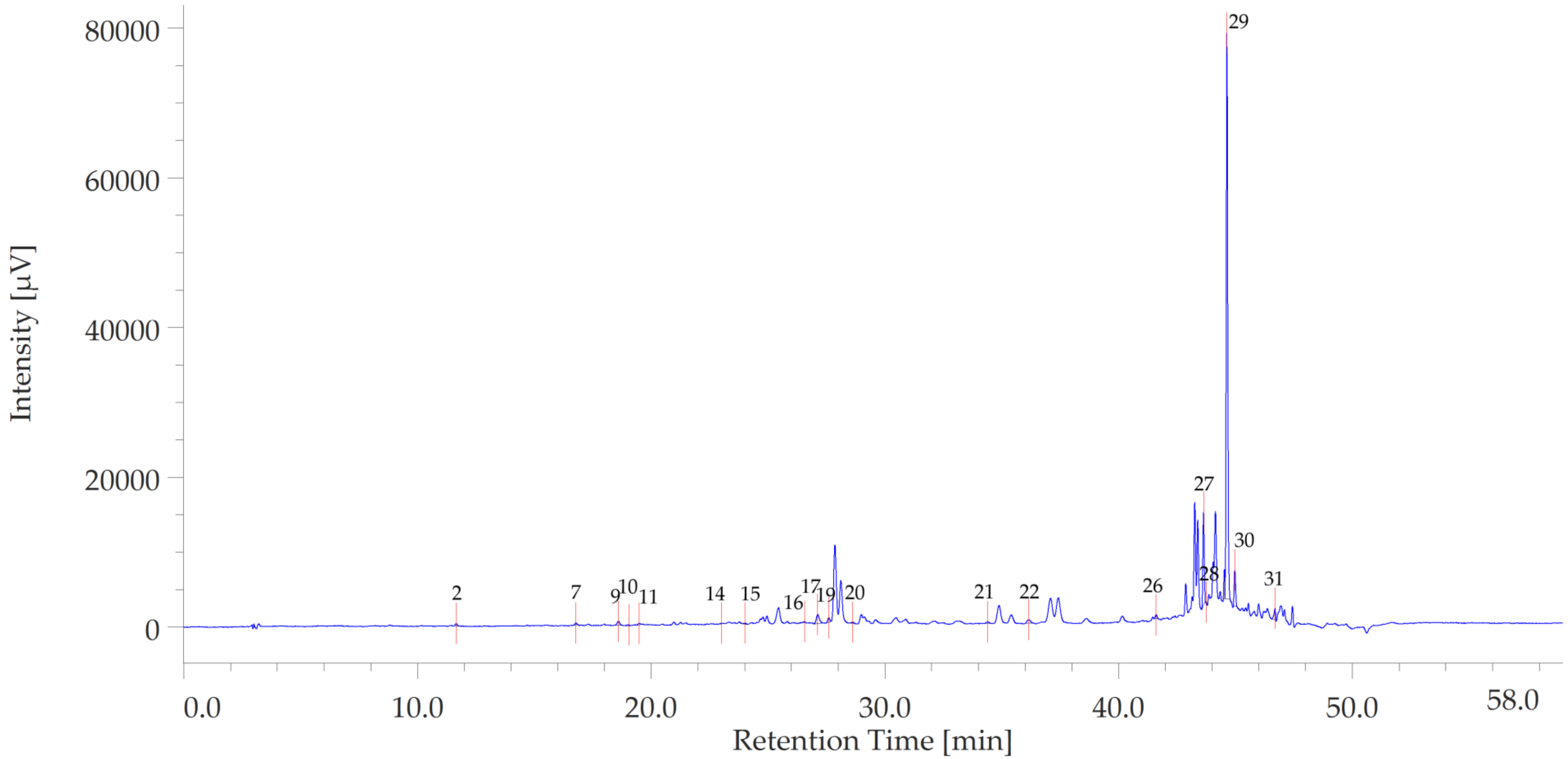

Intensity [µV]
80000
60000
40000
20000
0
0.0
10.0
20.0
30.0
40.0
50.0
58.0
Retention Time [min]
2
7
9
10
11
14
15
16
17
19
20
21
22
26
27
28
29
30
31

**Dichloromethane (B)**

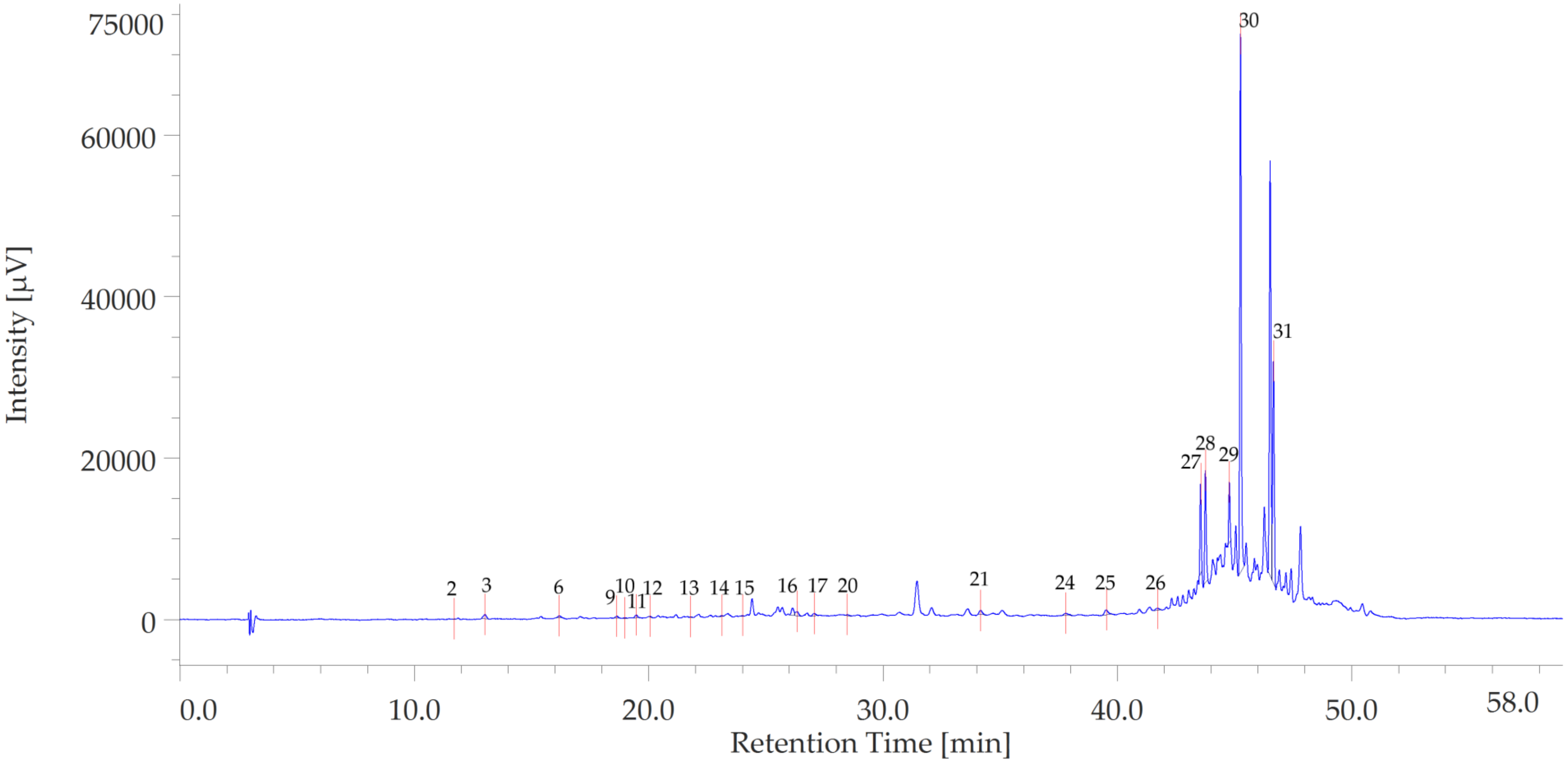

**Ethyl acetate (C)**

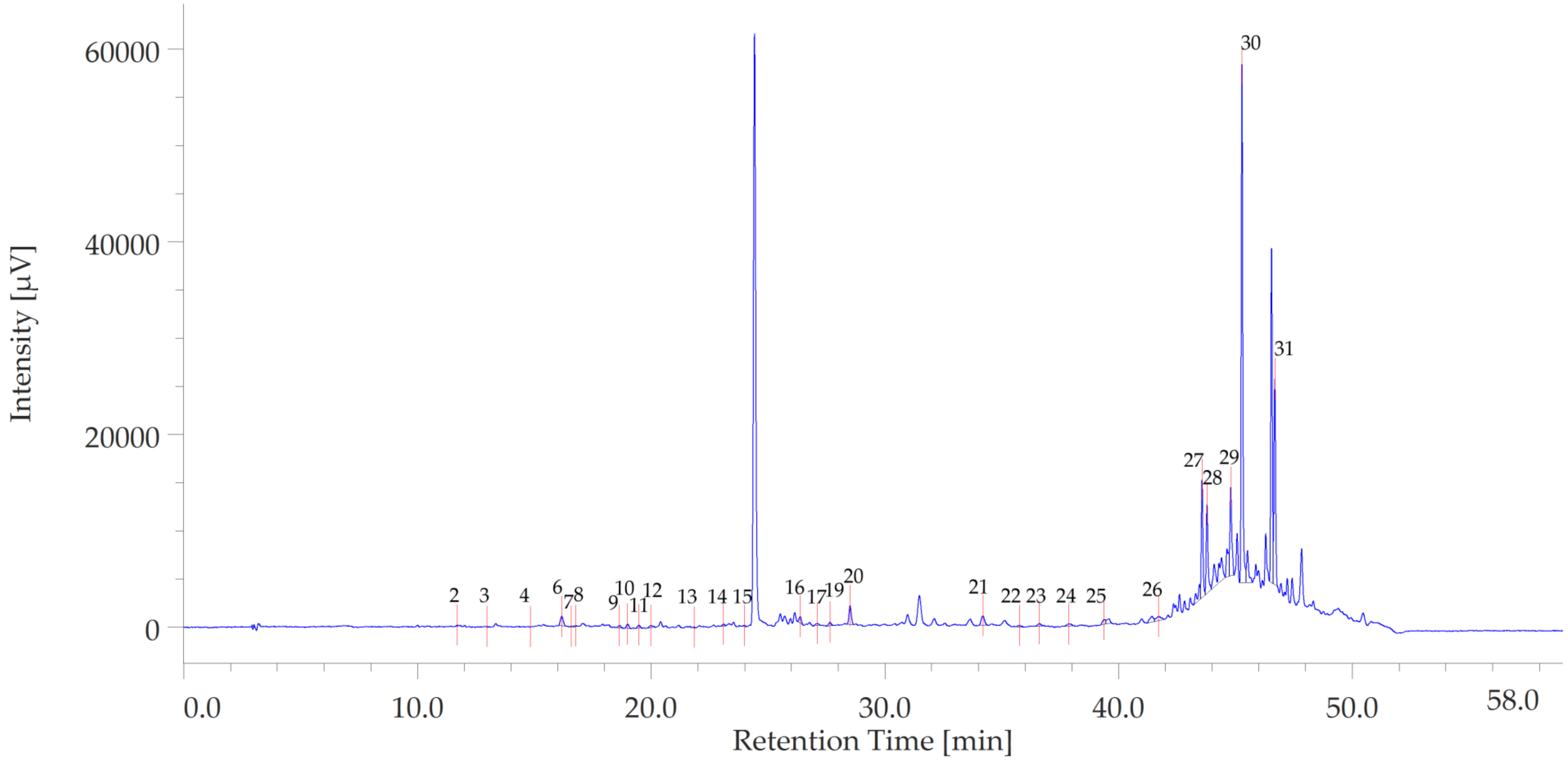

**Ethanol (D)**

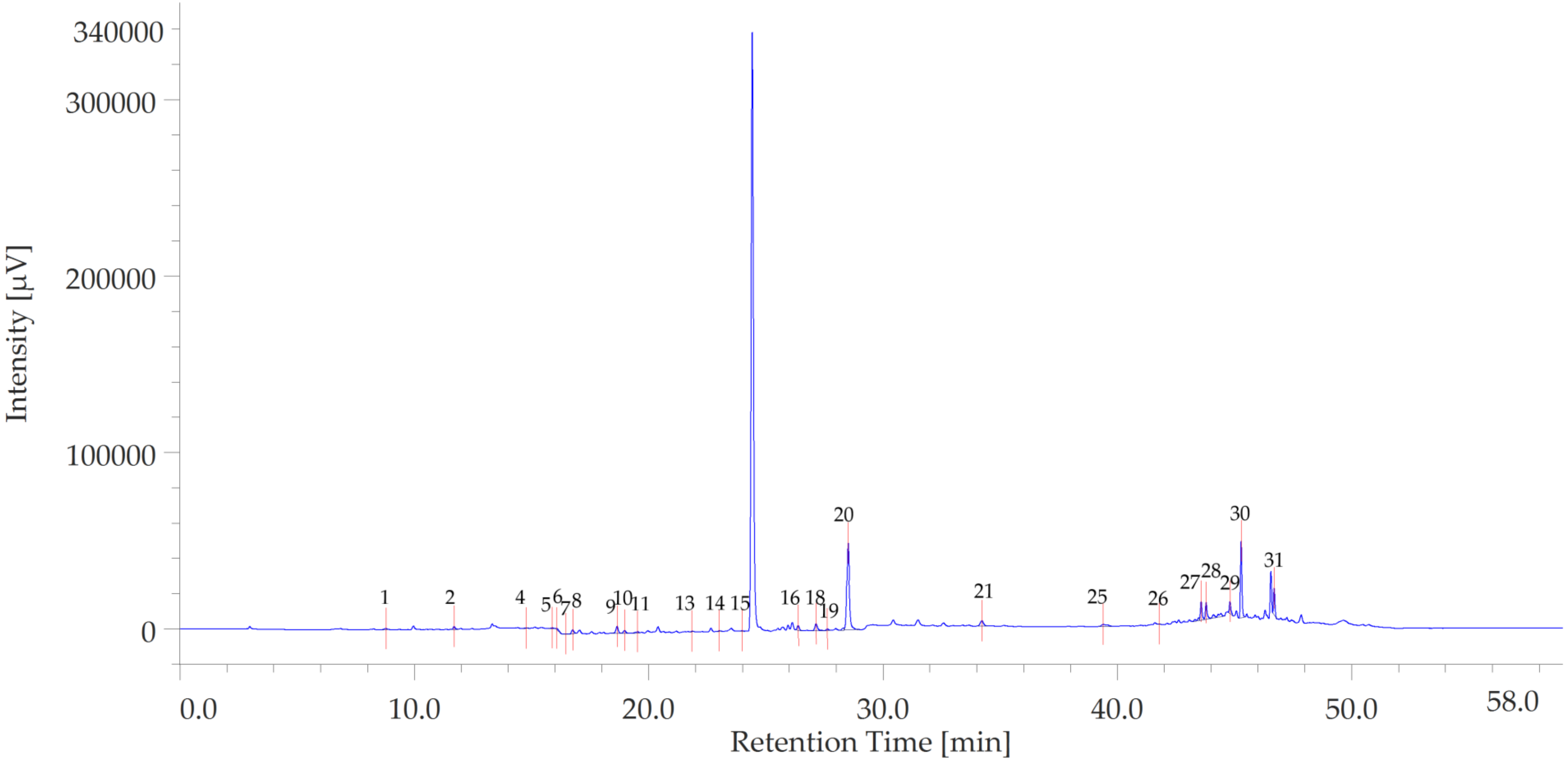

Intensity [µV]
340000
300000
200000
100000
0
Retention Time [min]
0.0
10.0
20.0
30.0
40.0
50.0
58.0
1
2
4
5
6
7
8
9
10
11
13
14
15
16
18
19
20
21
25
26
27
28
29
30
31

**Ethanol/ water (E)**

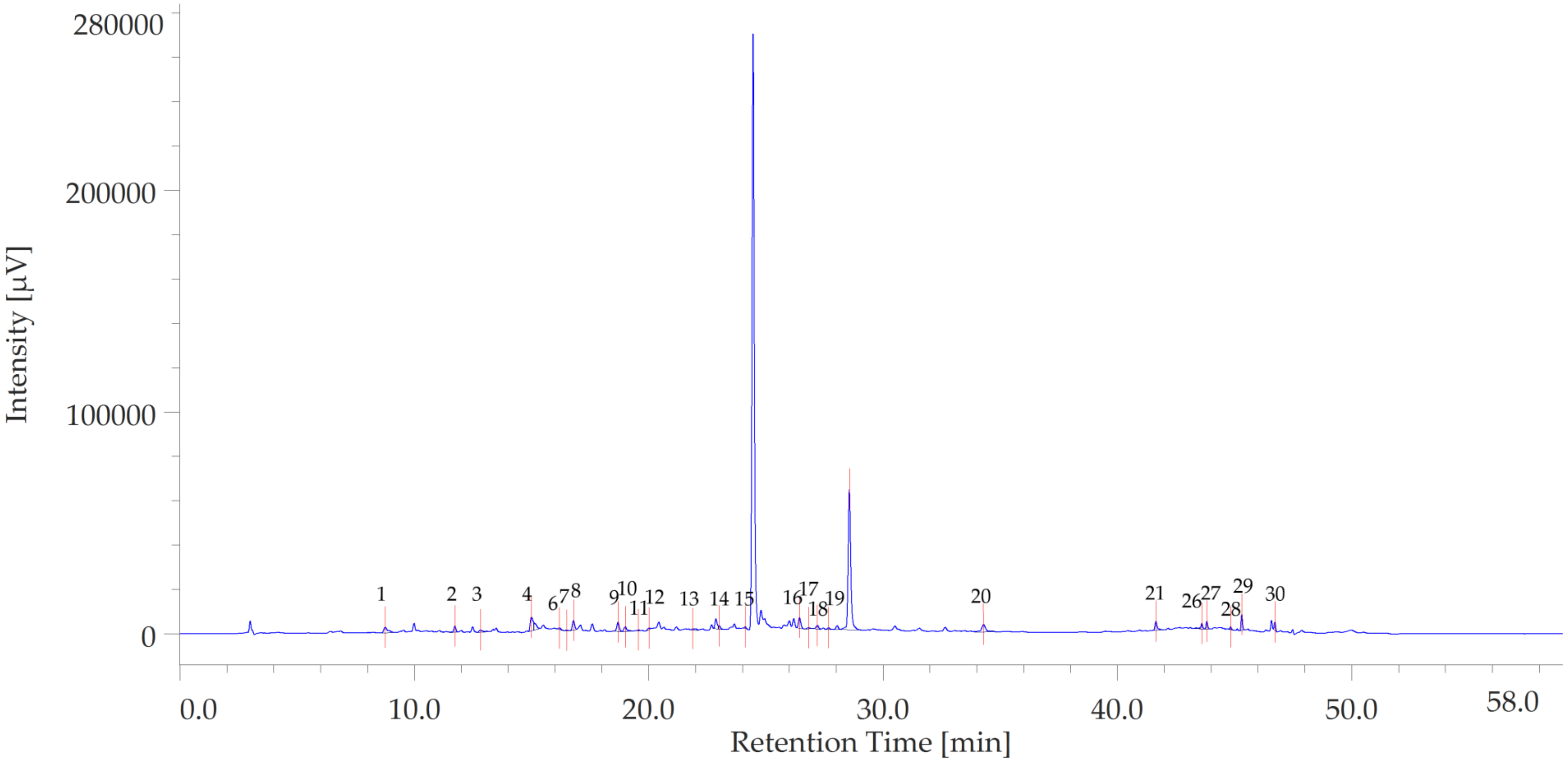

**Infusion (F)**

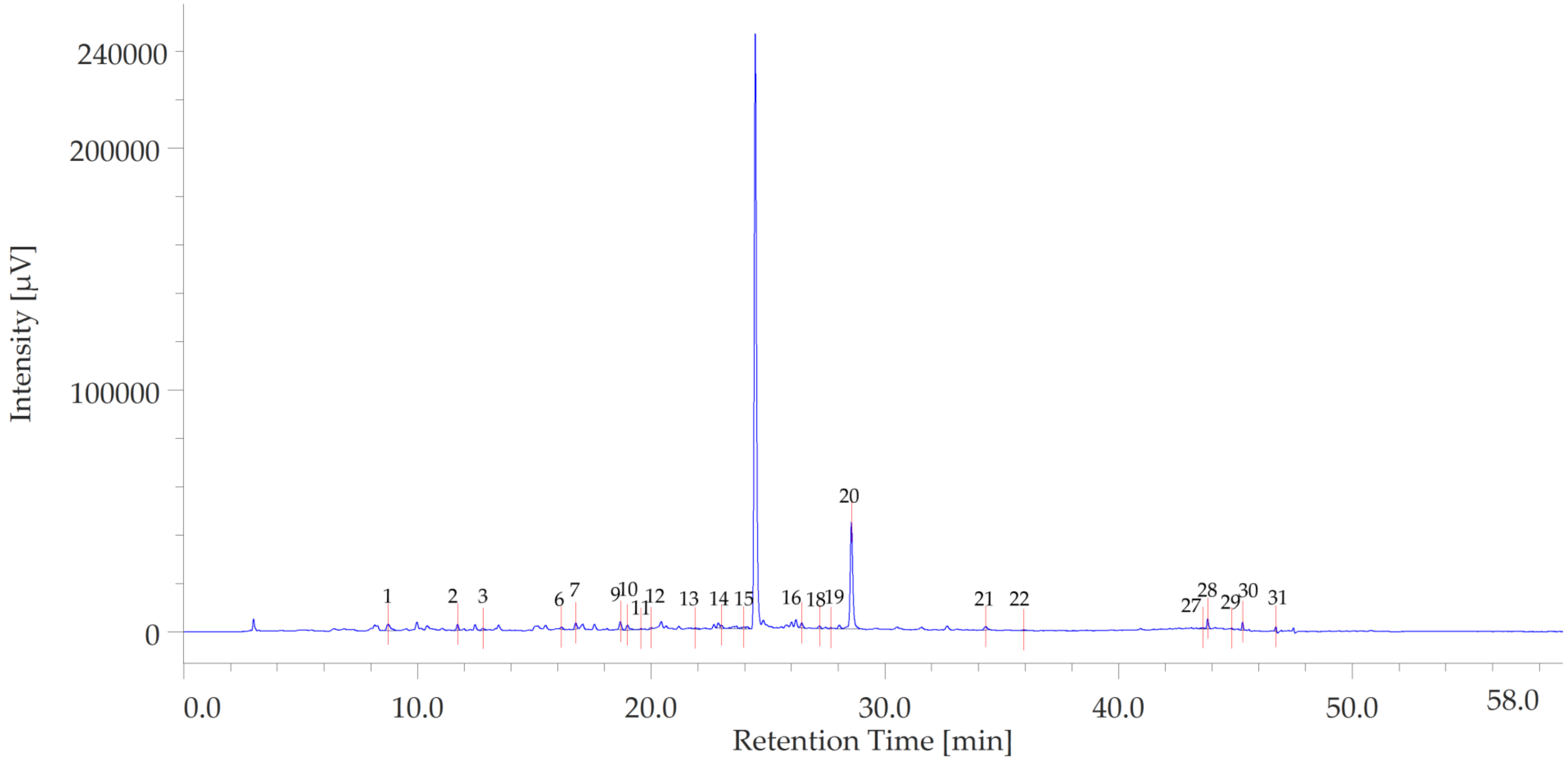


**Figure S1.** Representative chromatograms of the hexane (A), dichloromethane (B) ethyl acetate (C), ethanol (D), ethanol-water (E), and water (F) extracts from the aerial portions of *Nepeta italica*.

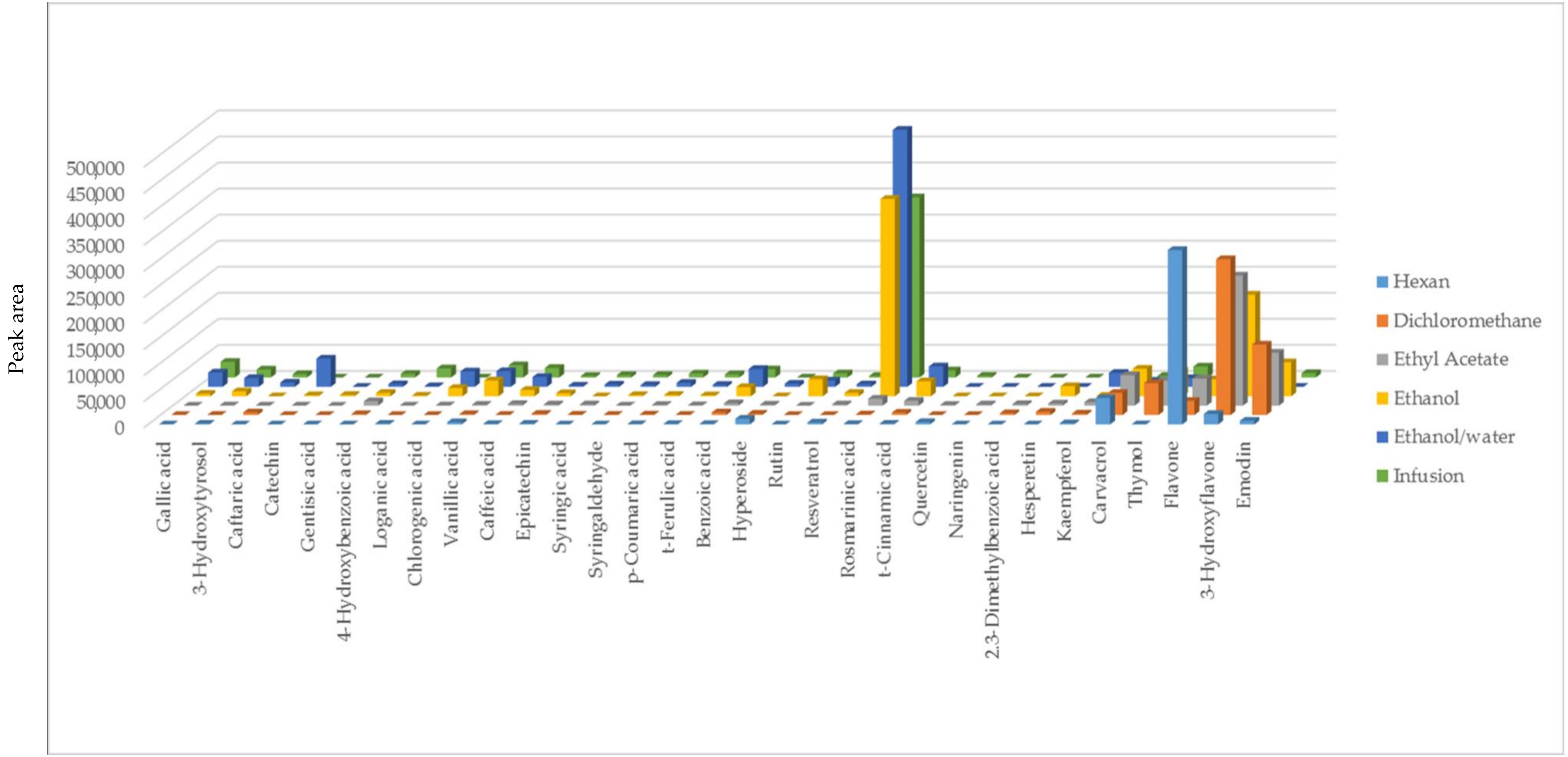


**Figure S2.** Phenolic profile of the *Nepeta italica* extracts.

**Ex Vivo Study**

Adult C57/BL6 male mice (3-month-old, weight 20–25 g) were housed in plexiglass cages (2–4 animals per cage; 55 cm × 33 cm × 19 cm) and maintained under standard laboratory conditions (21 ± 2 °C; 55 ± 5% humidity) on a 14/10 h light/dark cycle, with ad libitum access to water and food. Isolated colon specimens were collected form euthanized mice [Project no. F4738.N.5QP] and maintained in a humidified incubator with 5% CO2 at 37 °C for 4 h (incubation period), in RPMI buffer with added bacterial LPS (10 µg/mL), as previously described (Recinella et al., 2022). During the incubation period, the tissues were exposed to the extract (10-100 µg/mL).

**RNA Extraction, Reverse Transcription Andreal-Time Reverse Transcription Polymerase Chain Reaction (RT-PCR)**

Total RNA was extracted from the mouse colon specimens using TRI Reagent (Sigma–Aldrich, Milano, Italy), according to the manufacturer's protocol. Contaminating DNA was removed using 2 units of RNase-free DNase 1 (DNA-free kit, Ambion, Austin, TX, USA). The RNA concentration was quantified at 260 nm by spectrophotometer reading (BioPhotometer, Eppendorf, Hamburg, Germany), and its purity was assessed by the ratio at 260 and 280 nm readings. The quality of the extracted RNA samples was also determined by electrophoresis through agarose gels and staining with ethidium bromide, under UV light. One microgram of total RNA extracted from each sample in a 20 µL reaction volume was reverse transcribed using High Capacity cDNA Reverse Transcription Kit (Thermo Fisher Scientific, Waltham, MA, USA). Reactions were incubated in a 2720 Thermal Cycler (Thermo Fisher Scientific, Waltham, MA, USA) initially at 25 °C for 10 min, then at 37 °C for 120 min and finally at 85 °C for 5 s.

Gene expression of COX-2 and TNF-α were determined by quantitative real-time PCR using TaqMan probe-based chemistry, as previously described. PCR primers and TaqMan probes were purchased from Thermo Fisher Scientific Inc. The Assays-on-Demand Gene Expression Products used for gene expression evaluations in the mouse colon specimens were Mm00478374_m1 for COX-2 gene, Mm00443258_m1 for TNF-α gene, Mm00607939_s1 for β-actin gene. β-actin was used as the house-keeping gene. The elaboration of data was conducted with the Sequence Detection

System (SDS) software version 2.3 (Thermo Fischer Scientific). Relative quantification of gene ex-pression was performed by the comparative $2^{-\Delta\Delta Ct}$ method (Livak and Schmittgen, 2001).

Chiavaroli, A., Libero, M.L., Di Simone, S.C., Acquaviva, A., Recinella, L., Leone, S., Brunetti, L., Cicia, D., Izzo, A.A., Orlando, G., 2023. Adding New Scientific Evidences on the Pharmaceutical Properties of Pelargonium quercetorum Agnew Extracts by Using In Vitro and In Silico Approaches. Plants 12, 1132.

El-Moataz Bellah El-Naggar et al. Artemisinin from minor to major ingredient in *Artemisia annua* cultivated in Egypt. J App Pharm Sci, 2013; 3 (08): 116-123.

Livak, K.J., Schmittgen, T.D., 2001. Analysis of relative gene expression data using real-time quantitative PCR and the 2− ΔΔCT method. methods 25, 402-408.

Recinella, L., Chiavaroli, A., Veschi, S., Cama, A., Acquaviva, A., Libero, M.L., Leone, S., Di Simone, S.C., Pagano, E., Zengin, G., 2022. A grape (*Vitis vinifera* L.) pomace water extract modulates inflammatory and immune response in SW-480 cells and isolated mouse colon. Phytotherapy Research 36, 4620-4630.
